# Immunomodulatory Effects of BRAF, MEK, and CDK4/6 Inhibitors: Implications for Combining Targeted Therapy and Immune Checkpoint Blockade for the Treatment of Melanoma

**DOI:** 10.3389/fimmu.2021.661737

**Published:** 2021-05-07

**Authors:** Emily J. Lelliott, Grant A. McArthur, Jane Oliaro, Karen E. Sheppard

**Affiliations:** ^1^ Cancer Research Division, Peter MacCallum Cancer Centre, Melbourne, VIC, Australia; ^2^ Sir Peter MacCallum Department of Oncology, University of Melbourne, Parkville, VIC, Australia; ^3^ Department of Immunology, Central Clinical School, Monash University, Melbourne, VIC, Australia; ^4^ Department of Biochemistry and Pharmacology, University of Melbourne, Parkville, VIC, Australia

**Keywords:** melanoma, targeted therapeutic drugs, immunotherapy, CDK4/6 inhibition, BRAF/MEK inhibition, immune checkpoint blockade

## Abstract

The recent advent of targeted and immune-based therapies has revolutionized the treatment of melanoma and transformed outcomes for patients with metastatic disease. The majority of patients develop resistance to the current standard-of-care targeted therapy, dual BRAF and MEK inhibition, prompting evaluation of a new combination incorporating a CDK4/6 inhibitor. Based on promising preclinical data, combined BRAF, MEK and CDK4/6 inhibition has recently entered clinical trials for the treatment of BRAF^V600^ melanoma. Interestingly, while BRAF- and MEK-targeted therapy was initially developed on the basis of potent tumor-intrinsic effects, it was later discovered to have significant immune-potentiating activity. Recent studies have also identified immune-related impacts of CDK4/6 inhibition, though these are less well defined and can be both immune-potentiating and immune-inhibitory. BRAF^V600^ melanoma patients are also eligible to receive immunotherapy, specifically checkpoint inhibitors against PD-1 and CTLA-4. The immunomodulatory activity of BRAF/MEK-targeted therapies has prompted interest in combination therapies incorporating these with immune checkpoint inhibitors, however recent clinical trials investigating this approach have produced variable results. Here, we summarize the immunomodulatory effects of BRAF, MEK and CDK4/6 inhibitors, shedding light on the prospective utility of this combination alone and in conjunction with immune checkpoint blockade. Understanding the mechanisms that underpin the clinical efficacy of these available therapies is a critical step forward in optimizing novel combination and scheduling approaches to combat melanoma and improve patient outcomes.

## Introduction

Melanoma is the deadliest and most aggressive form of skin cancer, predominately arising from exposure to damaging ultraviolet radiation (1–3). Early surgical resection of the primary lesion results in high survival rates (4, 5) but an undetected melanoma that metastasizes to secondary organs leads to poor patient prognosis. Prior to the advent of modern therapies, patients presenting with advanced metastatic melanoma had a median overall survival of 5.1 months from diagnosis, with a 3-year survival probability of less than 5% (6). However, in 2011, the development of two new classes of therapeutics revolutionized the landscape of melanoma treatment; targeted therapies and immunotherapies. While unrelated and mechanistically distinct, the unprecedented success of these two therapies in the clinic rapidly led to their integration as a standard-of-care treatment for melanoma patients. However, despite these remarkable therapeutic advances, the overall prognosis for patients with late stage melanoma remains poor, reflecting the limitations of these therapies.

While targeted therapies improve survival in almost all patients treated, many do not receive long-term benefits due to the eventual emergence of drug resistance (7–9). Combinatorial targeted therapy strategies are therefore being developed with the goal of overcoming this acquired resistance. However, tumors are inherently highly adaptable and targeting tumor-intrinsic pathways alone rarely leads to durable tumor regression. Immunotherapies, on the other hand, engage the host immune response, and the immune system is arguably the only anti-cancer tool that is equally as adaptable as the tumor itself. This is reflected in the clinical response to immune checkpoint blockade (ICB), where approximately 75% of patients who initially respond to this therapy achieve long-term sustained tumor regression (10–12). However, in contrast to targeted therapy, only a subset of patients respond to ICB (13–15). Additionally, complications arising from adverse events (16, 17), along with the high cost of administering these therapies (18), highlights an urgent need to better understand and predict which patients are most likely to respond, so that therapy choice and timing can be personalized.

The short-lived responses to targeted therapy and the lower initial response rates to ICB means that many melanoma patients inevitably receive both of these therapeutic classes at some point throughout the duration of their treatment. For example, a patient who progresses on targeted therapy may be switched to ICB as a second line of treatment, or vice versa. Interestingly, while the clinical success of targeted therapies is attributable to tumor-intrinsic inhibitory mechanisms, it is now clear that these therapies also modulate anti-tumor immunity, which can substantially impact on the concurrent or subsequent response to immunotherapy. The immunomodulatory activity of targeted therapies, and the potential of combined targeted therapy and ICB approaches will be discussed in this review.

## Targeted Therapies In Melanoma

Targeted therapies, as the name suggests, are designed to precisely target and inhibit tumor-intrinsic aberrant signaling pathways that drive cell survival and proliferation. Melanoma predominately arises through the accumulation of genetic mutations in a type of pigment-producing skin cell, called a melanocyte, induced by repeated exposure to UV radiation or other environmental factors (2, 3, 19). When genetic mutations arise in genes encoding proteins critical for the regulation of cell survival or entry into the cell cycle, uncontrolled cell proliferation and malignant transformation can occur (20). These ‘oncogenic driver mutations’ result in constitutive activation of growth signaling pathways that are responsible for both the initiation of tumorigenesis and the maintenance of cancer growth. In a concept known as ‘oncogene addiction’, malignant cells become reliant on these aberrant signaling pathways (21), and as a result, blocking these pathways with targeted small molecule inhibitors can profoundly disrupt cancer progression through the rapid induction of tumor cell cytostasis or death. Here we focus on three targeted therapies for the treatment of metastatic melanoma, namely BRAF, MEK and CDK4/6 inhibitors.

### Inhibitors of the MAPK/ERK Pathway

**Figure 1:**
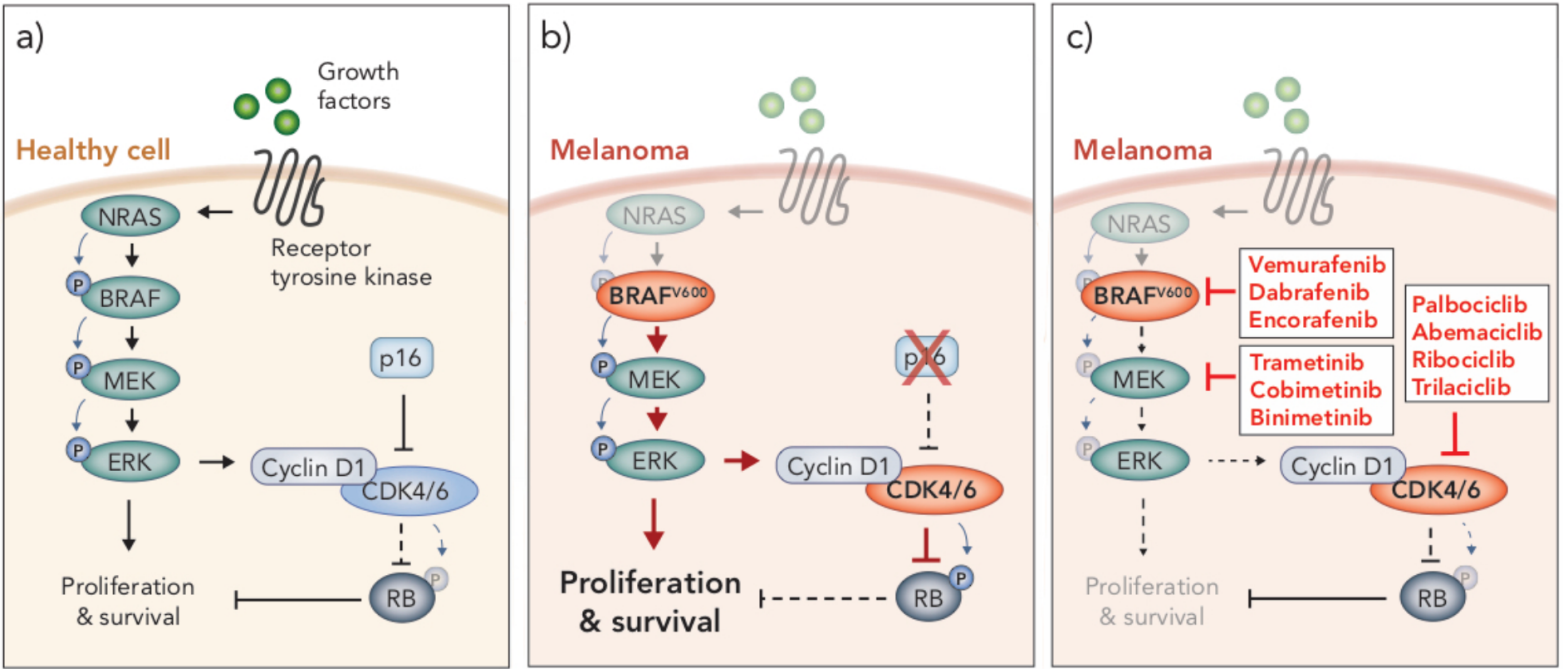
MAPK/ERK and p16/CyclinD-CDK4/6/RB signaling pathways in health and disease. **(A)** In healthy cells, growth factors bind receptor tyrosine kinases on the surface of the cell to stimulate proliferation and survival through the MAPK/ERK signaling pathway, which is mediated through a cascade of phosphorylation events (NRAS → BRAF → MEK → ERK). MAPK/ERK signaling promotes cyclin D1-CDK4/6 complex formation. Inhibition of CDK4/6 by p16 prevents CDK4/6-mediated phosphorylation of RB, thereby allowing RB to carry out its tumor suppressive function. **(B)** BRAF^V600^ mutations and loss of p16 are often seen in melanoma. BRAF^V600^ is constitutively active and promotes aberrant overactivation of MAPK/ERK signaling. In the absence of p16, CDK4/6 phosphorylates and inhibits the tumor suppressor RB. These events lead to uncontrolled cellular proliferation and survival. **(C)** Clinical inhibitors against BRAF^V600^, MEK and CDK4/6 block MAPK/ERK signaling and CDK4/6-mediated suppression of RB, thereby attenuating cell cycle and survival.

A number of oncogenic driver mutations are found in melanoma, though the most common occur in the gene encoding the protein kinase BRAF. This mutation occurs at codon 600, resulting in substitution of a valine (V) residue, often with a glutamate (E), which renders the kinase constitutively active (22–24). This BRAF^V600E^ mutation occurs in around 40-50% of melanomas (25) and leads to constitutive activation of the mitogen-activated protein kinase/extracellular signal-regulated kinase 1/2 (MAPK/ERK) signaling pathway (22–24) (**Figure 1**). This pathway is a critical regulator of cell cycle, survival and differentiation, and is typically activated only in the presence of growth factors, which bind receptor tyrosine kinase receptors on the cell surface, initiating a tightly controlled cascade of kinase-mediated activation events (RAS→RAF→MEK→ERK) (26) (**Figure 1**). However, as in the case of BRAF^V600^ melanoma, aberrant activation of MAPK/ERK signaling is observed in a number of cancers due to dysregulation of one of the protein components in the pathway (26, 27). Indeed, in melanoma other oncogenic driver mutations mediate tumorigenesis *via* activation of MAPK/ERK signaling, including activating NRAS mutations and loss-of function mutations in NF1, a GTPase known to downregulate RAS activity (28). NRAS and NF1 mutations are less frequent than BRAF mutations, occurring in approximately 10-25% and 14% of melanomas, respectively (28). Interestingly, driver mutations that mediate activation of this pathway can co-exist in the same tumor but are mutually exclusive at a single cell level (29), indicating that a single MAPK/ERK activation event is sufficient to drive oncogenesis. Given the frequency of aberrant MAPK/ERK activity observed in melanoma, several inhibitors have been developed to target various components of this pathway, including inhibitors of BRAF, MEK and more recently RAS and ERK (reviewed in 30, 31). Here, we focus on BRAF and MEK inhibitors, and their use in treating the most prevalent form of melanoma, BRAF^V600^ melanoma.

Small molecule inhibitors of mutant BRAF were developed to block protein kinase activity and prevent activation of MAPK/ERK signaling in tumor cells (32) (**Figure 1**). Remarkably, in a phase III clinical trial, the first BRAF inhibitor, vemurafenib, showed such extraordinary success that the trial was stopped prematurely so that patients in the alternative trial arm could access the treatment (33). Vemurafenib was subsequently FDA approved for the treatment of BRAF^V600^ melanoma in 2011, followed by two more BRAF inhibitors (dabrafenib and encorafenib) in the years following (34, 35). The clinical response to BRAF inhibition was typified by rapid and robust tumor regression in almost all patients treated, with very minimal toxicities (33). However, those responses were short-lived, as tumors rapidly developed resistance to these drugs (33, 36). Resistance has been shown to occur through various genomic and transcriptional alterations that lead to MAPK/ERK reactivation, as well as activation of alternative pathways involved in growth and survival (reviewed in ref. 36). Nonetheless, BRAF inhibitors improved median progression-free survival (PFS) from 1.6 months (on the standard chemotherapy, dacarbazine) to 5.3 months (33); a momentous survival benefit for a disease in which no progress had been made for decades.

The predominant mechanism of resistance to BRAF inhibitors is reactivation of MAPK/ERK signaling (36, 37). As such, shortly after BRAF inhibitors entered the clinic, trials commenced to examine the efficacy of combining BRAF inhibition with inhibition of its downstream substrate kinase MEK, with the goal of overcoming MAPK/ERK reactivation (38, 39) (**Figure 1**). This combination demonstrated considerable success, improving median PFS by a further 5 months compared to BRAF inhibition alone, and still with minimal toxicities (38, 39). As such, numerous MEK inhibitors have been developed for clinical use (e.g. trametinib, cobimetinib, binimetinib) and dual BRAF and MEK inhibition is now considered the standard-of-care over BRAF inhibitor monotherapy for the treatment of BRAF^V600^ melanoma patients. However, similar to BRAF inhibitor monotherapy, the majority of patients treated with dual BRAF and MEK inhibitors inevitably develop resistance less than a year into treatment (38, 39). As such, further clinical trials are ongoing to examine other molecular targets to treat this disease.

### Inhibitors of CDK4/6

An emerging target in melanoma is the cyclin-dependent kinases 4 and 6 (CDK4/6). These kinases, which are regulated by type D cyclins, phosphorylate and inhibit the tumor suppressor retinoblastoma protein (RB) (40) (**Figure 1**). RB is a central regulator of G1-S cell cycle transition (41, 42). In its hypophosphorylated form, RB binds to and inhibits E2F transcription factors, preventing transcription of genes associated with cell cycle progression and entry into S phase (41, 43, 44). Hence, CDK4/6 plays a critical role in cell cycle regulation in G1, through mediation of the phosphorylation status and subsequent function of RB (45, 46). Aberrant activity of CDK4/6 leads to hyperphosphorylated RB, E2F-mediated progression through G1 and uncontrolled cell proliferation – a hallmark of cancer (47). Overactivity of CDK4/6 is commonly due to an inactivating mutation or deletion in the *CDKN2A* gene which encodes the CDK4/6 inhibitor, p16^INK4a^ (48) (**Figure 1**). The p16^INK4a^/CyclinD-CDK4/6/RB axis is dysregulated in numerous cancers, including around 90% of melanomas (49, 50). In many cancers, this is due to loss of function of RB itself, however, in melanoma RB is often functional, and instead aberrant activation of this pathway is predominately driven by a loss of p16^INK4a^ and, subsequently, constitutive CDK4/6 activity (51). Interestingly, the functional loss of p16^INK4a^ and RB appear to be mutually exclusive events in this pathway (44, 47, 52), similar to the activating mutations in MAPK/ERK signaling.

Augmented CDK4/6 activity is observed in many cancers in addition to melanoma, making it an attractive therapeutic target for the treatment of a variety of malignancies (41, 44, 53). The CDK4/6 inhibitor, palbociclib, entered its first clinical trial in 2011 for advanced solid tumors or non-Hodgkins lymphoma and was generally well tolerated (54). Importantly, only patients with tumor types in which RB was functional were recruited to these trials, as CDK4/6 inhibitors rely on the de-repression of functional RB to inhibit E2F and cell cycle progression. Following promising clinical outcomes, palbociclib was FDA approved for the treatment of HR-positive, HER2-negative breast cancer in 2015, irrespective of CDK4/6 alteration status (55). Two other CDK4/6 inhibitors (ribociclib, and abemaciclib) have since been FDA approved and, along with a fourth inhibitor (trilaciblib), continue to be evaluated in numerous clinical trials for various other malignancies, including metastatic melanoma (reviewed in ref. 56).

### Combined Inhibition of BRAF, MEK and CDK4/6

Downstream of MAPK/ERK signaling, activated ERK translocates to the nucleus and activates a suite of transcription factors (e.g. ETS, ELC1, MYC, STAT1/3), mediating a network of transcriptional changes that promote cell growth, survival and differentiation (57). Importantly, one of the secondary response genes induced by MAPK/ERK signaling is *CCND1*, which encodes the CDK4/6 binding partner Cyclin D1 (58, 59). MAPK/ERK signaling also functions post translationally to mediate assembly of the CyclinD-CDK4/6 complex (60). Hence, MAPK/ERK signaling impinges on the p16^INK4a^/CyclinD-CDK4/6/RB axis downstream (**Figure 1**). Accordingly, aberrant activation of the latter is associated with resistance to MAPK/ERK inhibition. Specifically, elevated expression of *CCND1* is associated with resistance to BRAF inhibition in BRAF^V600E^ melanoma, particularly when CDK4 is also overexpressed (61), and a loss of *CDKN2A* expression (indicative of elevated CDK4/6 activity) correlates with poor progression free survival in patients treated with dual BRAF and MEK inhibition (62). Together these observations make CDK4/6 an attractive kinase to target in combination with MAPK/ERK inhibitors. Indeed, a number of preclinical studies have reported synergy of CDK4/6 inhibition with BRAF/MEK inhibitors (63–65). In these studies, combined BRAF and CDK4/6 inhibition overcomes resistance to BRAF inhibitor monotherapy *in vitro* and *in vivo* in pre-clinical xenograft models of human melanoma. The triple combination has also demonstrated significantly enhanced efficacy compared to dual BRAFi/MEKi *in vitro* and *in vivo* (66). Dual inhibition of MEK and CDK4/6 has also shown promise in pre-clinical studies evaluating tumors with elevated MAPK/ERK signaling that is mediated through mechanisms other than mutant BRAF, such as mutations in RAS. For example, this combination has shown synergy in preclinical KRAS mutant colorectal cancer models (67), and is efficacious against a subset of NRAS and other melanoma subtypes (68–70). Indeed, early clinical trials of dual MEK and CDK4/6 inhibition in NRAS mutant melanoma demonstrated encouraging results (NCT01719380). A phase Ib/II clinical trial examining the safety and efficacy of combination BRAF and CDK4/6 inhibition was terminated early, but showed the combination was generally well tolerated (NCT01777776). Despite promising preclinical data, in a phase Ib/II dose escalation study, the triple combination of encorafenib (BRAFi), binimetinib (MEKi) and ribociclib (CDK4/6i) did not improve response rates or PFS compared to dual encorafenib/binimetinib (71). A further dose escalation phase Ib trial is ongoing to assess the triple combination of encorafenib, binimetinib and palbociclib in patients naïve to or with resistance to BRAF/MEK inhibitors (NCT04720768). Based on limited clinical data, it is possible this triple combination may not be as potent as first hoped from the preclinical evidence of tumor-intrinsic activity. As such, a better understanding of the impact of CDK4/6i on BRAFi/MEKi-mediated immune enhancement is critical for understanding the path forward with this combination, both as a stand-alone therapy in regard to optimal sequencing of these inhibitors, as well as their potential to be combined with ICB.

## Immune Checkpoint Inhibitors

In 2011, not only was the first targeted therapy approved for the treatment of melanoma, so too was the first field-changing immunotherapy, ipilimumab. In contrast to targeted therapies, which aim to directly inhibit tumor growth by blocking tumor-intrinsic pathways, immune checkpoint inhibitors aim to activate the host immune system to control and eradicate cancer. Ipilimumab is a monoclonal antibody developed against the immune checkpoint inhibitory molecule, CTLA-4. Immune checkpoints are regulatory mechanisms that are essential to maintain immune homeostasis and fine-tune immune responses during infection. In the setting of tumor pathology however, engagement of immune checkpoints contributes to tumor immune evasion, and hence, blocking the activity of these receptors can improve anti-tumor immune responses. In clinical trials, ipilimumab significantly improved median overall survival (OS) from 9.1 months to 11.2 months compared to conventional chemotherapy, and most notably promoted durable tumor control, with significantly improved 3-year survival probability (13, 15). This led to FDA approval of ipilimumab for the treatment of metastatic melanoma in 2011.

In 2014, a second class of immune checkpoint inhibitors, PD-1 inhibitors, were FDA approved for use in patients with unresectable or metastatic melanoma (72, 73). Similar to ipilimumab, PD-1 inhibitors are also monoclonal antibodies that de-repress T cell immunity, but they achieve this through targeting the T cell inhibitory molecule, PD-1 (74). The discovery that PD-1 was a negative regulator of anti-tumor immunity led to a series of preclinical studies and clinical trials demonstrating that blockade of PD-1 was an effective therapy for the treatment of melanoma and other cancers (reviewed in 75, 76). In a seminal phase III clinical trial (CheckMate-066), the PD-1 inhibitor, nivolumab, significantly improved the overall rate of survival (72.9% vs. 65.5%), and median PFS of melanoma patients (5.1 months vs. 2.2 months) compared to chemotherapy, and similar to ipilimumab, promoted durable tumor control (14). Anti-PD-1 therapies also compared favorably to ipilimumab in regard to both efficacy and tolerability (11, 12, 77). More recent trials have shown superior anti-tumor efficacy with the dual combination of CTLA-4 and PD-1 blockade in melanoma, suggesting concurrent administration may be a beneficial therapeutic strategy; although with increased risk of toxicities (11, 78, 79). Overall, the advent of CTLA-4 and PD-1 immune checkpoint inhibitors dramatically shifted the field of cancer therapeutics and prompted the inclusion of immune evasion as a hallmark of cancer.

### Mechanisms Underpinning the Efficacy of Immune Checkpoint Blockade

CTLA-4 blockade has shown efficacy in a number of preclinical tumor models (reviewed in 80). While the primary mechanism is thought to be direct de-repression of CD8+ T cell activity by blocking CTLA-4 antagonism of the T cell costimulatory molecule CD28, recent studies have demonstrated that CTLA-4 blockade has multiple anti-tumor effects, including the reversal of T regulatory cell (Treg) mediated immunosuppression in the tumor microenvironment (81). CTLA-4 is constitutively expressed on Tregs and contributes to their regulatory and immunosuppressive action through multiple mechanisms (82, 83). CTLA-4 expression on Tregs is much higher than that of CD8^+^ T cells, and as a result, CTLA-4 antibodies bind Tregs in high amounts and consequently promote their depletion *via* natural killer (NK) cell-mediated antibody-dependent cellular cytotoxicity (84). Notably, while CTLA-4 blockade has shown efficacy in numerous mouse tumor models, this appears largely dependent on the stage of disease and tumor burden (85). In addition, less immunogenic cancers, including the B16 melanoma model, demonstrate limited responses (86).

Since the advent and ensuing success of anti-PD-1/PD-L1 as a cancer immunotherapy, the mechanisms of action continue to be explored. While originally thought to simply re-invigorate T cells with high expression of PD-1 within the tumor microenvironment, a recent study found that the success of anti-PD-1 therapy relied on the clonal replacement of these cells through the recruitment of new T cells into the tumor microenvironment (87). Indeed, evidence suggests that myeloid-derived T cell chemoattractants are required for anti-PD-1 efficacy (88, 89), further supporting the importance of T cell recruitment for the success of this immunotherapy. Furthermore, tumor infiltrating T cells exist across a spectrum of functional and dysfunctional phenotypic states, and it is proposed that these different states vary in their capacity to respond anti-PD-1 therapy.

Following the profound success of ICB in a subset of melanoma patients, thousands of clinical trials are now underway to test the efficacy of these checkpoint inhibitors either alone, or in combination with other anti-cancer agents, for the treatment of melanoma and a range of other cancers. Notably, utilizing existing therapeutics to sensitize tumors to ICB is a highly sought-after strategy. Indeed, the incorporation of ICB with targeted therapies for the treatment of melanoma is an emerging area of clinical interest given the considerable immunomodulatory activity of targeted therapies that has been observed in recent years. Such strategies, however, require a thorough understanding of these immunomodulatory effects, and this will be discussed in the following sections.

## Immunomodulatory Activity Of Targeted Therapies

While the rationale behind targeted therapies is to inhibit intrinsic mechanisms of tumorigenesis, many oncogenic targets, including BRAF, MEK and CDK4/6, are also involved in immune signaling pathways, in both malignant cells and healthy immune cells. Indeed, there is now considerable evidence implicating a role for host immunity in the efficacy of these targeted therapies, which will be the focus of the following sections.

### Immunomodulation by BRAF and MEK Inhibitors

**Figure 2:**
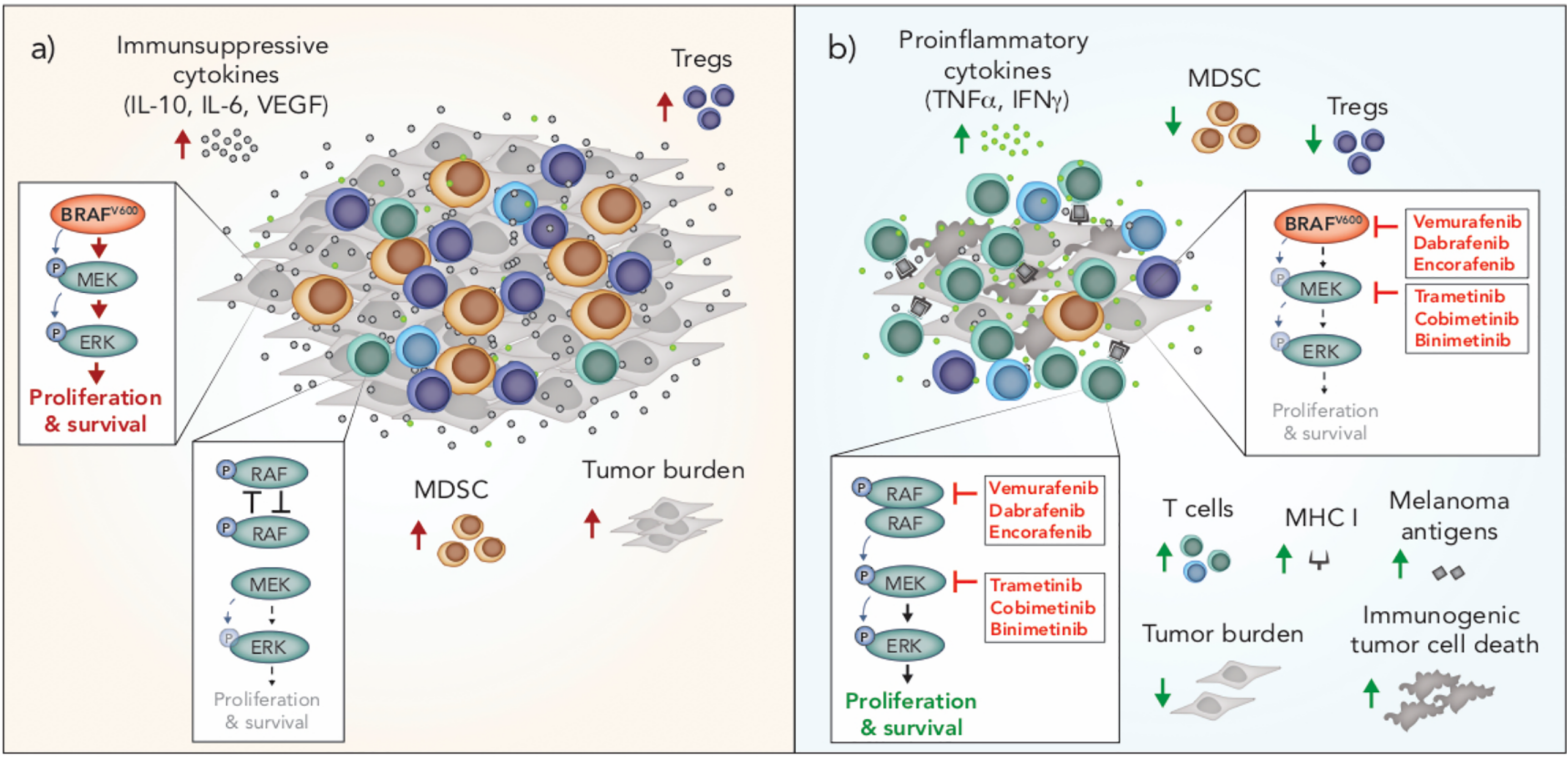
Immunomodulation by oncogenic BRAF and inhibitors of BRAF and MEK in melanoma. **(A)** BRAF^V600^ promotes tumor progression through tumor-intrinsic activation of MAPK/ERK signaling and increased production of immunosuppressive cytokines. MAPK/ERK signaling *via* oncogenic BRAF^V600^ is associated with increased frequencies of Tregs and MDSCs in the tumor microenvironment. In T cells, RAF is not overactive and there is no aberrant MAPK/ERK signaling. **(B)** BRAF and MEK inhibition blocks MAPK/ERK signaling in BRAF^V600^ tumor cells and induces immunogenic cell death. Inhibition of MAPK/ERK promotes the upregulation of MHC I and expression of melanoma associated antigens by tumor cells and is associated with increased frequencies of T cells and pro-inflammatory cytokines in the tumor microenvironment. BRAF inhibition is also associated with a reduction in intratumoral immunosuppressive cells including Tregs and MDSCs. In T cells and other cells with wild type BRAF, BRAF inhibition promotes paradoxical MAPK/ERK activation through RAF dimerization and dimer-dependent enzyme transactivation, enhancing the proliferation and survival of these cells. MDSC, myeloid derived suppressor cell; Treg, Regulatory T cell.

Tumors are notorious for evolving mechanisms to avoid immune surveillance, and oncogenic BRAF in melanoma cells can induce changes that facilitate tumor immune escape. For example, activated MAPK/ERK signaling *via* oncogenic BRAF is associated with tumor cell production of immunosuppressive cytokines (VEGF, IL-6, IL-10) and downregulation of MHC I, which leads to impairment of dendritic cell (DC) maturation, reduced T cell recognition and recruitment of suppressive myeloid-derived suppressor cells (MDSCs) and Tregs into the tumor microenvironment (90–92) (**Figure 2**). Conversely, inhibition of BRAF (BRAFi) is associated with significant immunological changes in the tumor microenvironment that are generally considered favorable for anti-tumor immunity. These changes include increased tumor infiltrating lymphocytes (TILs) (93–95), a higher ratio of cytotoxic T cells to regulatory T cells (93), up-regulation of MHC expression on tumor cells and enhanced presentation of melanoma-associated neo-antigens (91, 95, 96), increased production of IFN-γ and TNF-α (93) and reduced production of immunosuppressive cytokines, such as IL-6, IL-10 and VEGF (90, 95) **(** **Figure 2** **)**. Interestingly, resistance to BRAFi is associated with a reversal of these immune-potentiating effects, with a loss of TILs and induction of T cell exhaustion markers, including PD-1, observed in tumors that have progressed on BRAFi (93, 94).

While BRAF inhibition can result in favorable changes in the tumor immune microenvironment due to tumor intrinsic effects, these inhibitors also have direct effects on lymphocytes and other immune cells. In the absence of mutant BRAF, BRAFi has been shown to paradoxically activate the MAPK/ERK pathway by promoting RAF dimerization and dimer-dependent enzyme transactivation in immune cells (97–99) (**Figure 2**). This paradoxical activation has been observed in T cells (100), NK cells (101) and macrophages (102). MAPK/ERK paradoxical activation in T and NK cells is associated with a favorable anti-tumor response due to the resulting increase in proliferation of these cytotoxic lymphocytes. In contrast, paradoxical activation in macrophages leads to the production of VEGF, which promotes tumor growth and resistance to BRAF inhibition (102).

Unlike BRAFi, which are designed to selectively inhibit oncogenic BRAF in melanoma, MEK inhibition (MEKi) is designed to target wild type MEK, which is universal across melanoma and other cell types. In cells with wild type BRAF, the addition of a MEK inhibitor reduces BRAFi-induced MAPK/ERK paradoxical activation (103). Early *in vitro* studies of the effects of MEK inhibition on the immune system raised concerns that that this inhibitor would dampen the immune response to melanoma. Specifically, MEKi was shown to reduce T cell proliferation, the percentage of cytokine-producing T cells, antigen-specific T cell expansion and cross presentation by DCs (104). However, clinical results of combined BRAF and MEK inhibition were striking, not only due to significantly enhanced progression free survival, but also because the addition of a MEK inhibitor unexpectedly lowered the toxicity profile compared to BRAF inhibitor monotherapy (103); a phenomenon attributed to MEKi offsetting BRAFi-mediated paradoxical ERK activation in normal cells (103). Interestingly, MEKi also enhances the persistence of tumor infiltrating immune cells, prolonging anti-tumor T cell immunity (105), and may therefore delay or prevent the loss of T cells seen during the development of BRAFi resistance. BRAFi also causes an influx of regulatory T cells and myeloid suppressor cells into the tumor, and the addition of a MEK inhibitor appears to reverse this, leading to a more favorable tumor microenvironment (106) (**Figure 2**). Recently, dual BRAF and MEK inhibition was also shown to induce anti-tumor immunity *via* the induction of pyroptosis; a type of inflammatory cell death that promotes DC activation and subsequent T cell immunity (107). Interestingly, a number of melanoma patients treated with the combination of BRAFi and MEKi have achieved durable and ongoing tumor regression (7). Given the impact of these inhibitors on the immune system, it is prudent to question how much of this response is attributable to tumor-intrinsic effects, and how much is dependent on a favorable shift in anti-tumor immunity.

### Immunomodulation by CDK4/6 Inhibitors

Until recently, the immunomodulatory effects of CDK4/6 inhibition (CDK4/6i) were entirely unexplored. This changed rapidly in 2017 when a seminal study uncovered a role for CDK4/6i in augmenting anti-tumor immunity (108), prompting a spate of further studies in this area (109–112). Importantly, using various syngeneic mouse models, many of these studies showed that the efficacy of CDK4/6i is partially, or entirely abrogated, when the immune system is compromised. The immunomodulatory effects of CDK4/6i are multi-faceted and complex, and still not fully understood. Proposed mechanisms include both tumor-intrinsic effects, which indirectly modulate immunity, as well as direct effects on cells of the immune system, both of which likely contribute to significant remodeling of the tumor immune microenvironment.

#### Tumor-Intrinsic Immunomodulatory Effects of CDK4/6 Inhibition

**Figure 3:**
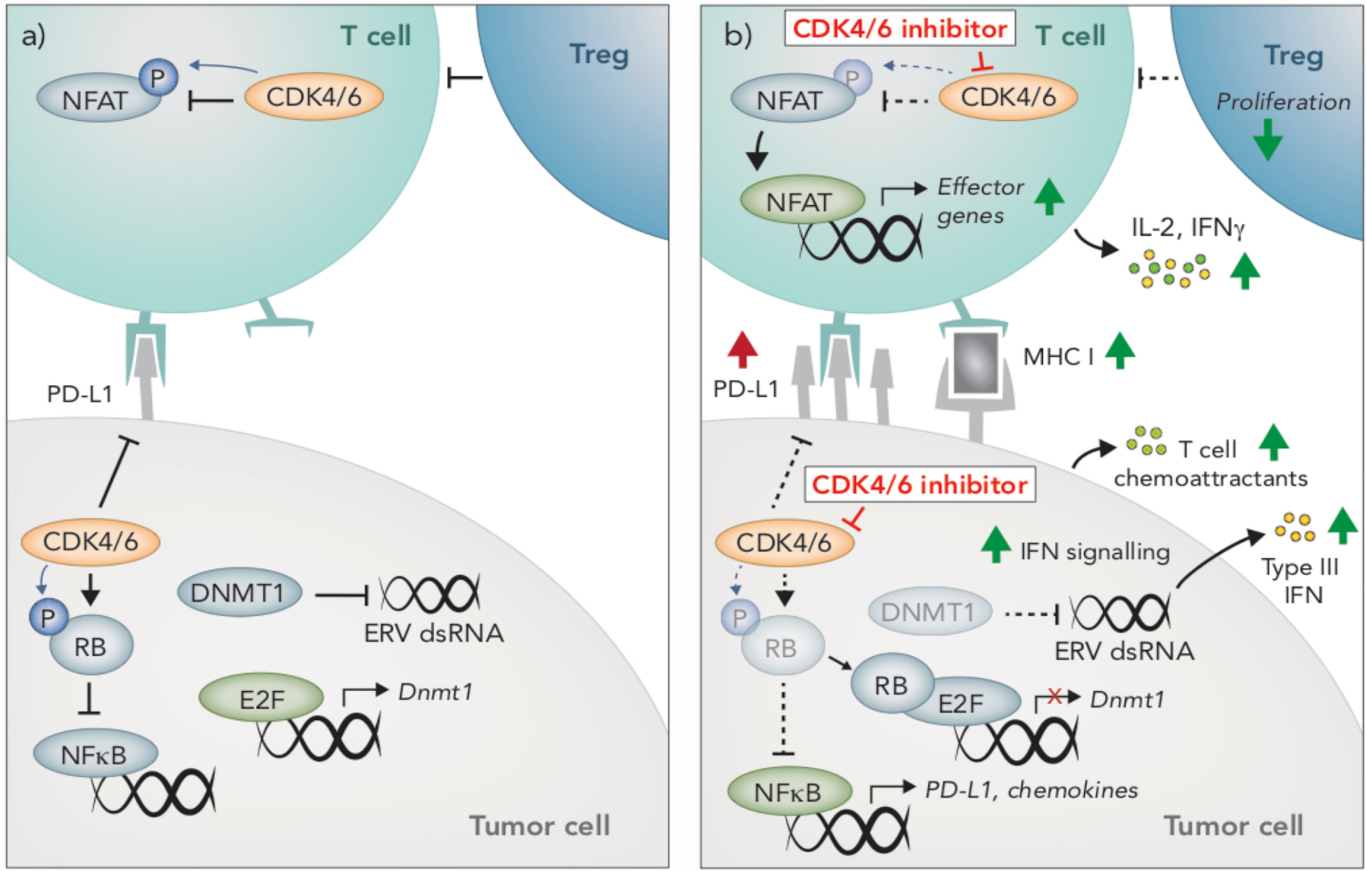
Immunomodulatory effects of CDK4/6 inhibition. **(A)** CDK4/6 inhibition modulates anti-tumor immunity through multiple mechanisms. In tumors cells, CDK4/6 inhibition leads to hypophosphorylated RB, which binds to and inhibits the activity of E2F transcription factors. Reduced E2F activity leads to an induction of Type III interferons (IFN), resulting in paracrine IFN signaling and upregulation of MHC I. This induction of IFN is due to the suppression DNMT1, (an E2F target gene), which reduces methylation of endogenous retroviral (ERV) genes, thereby promoting their expression and inducing viral mimicry. Hypophosphorylated RB also promotes activation of NFκB and subsequent upregulation of T cell chemoattractants and PD-L1. **(B)** CDK4/6 inhibition also prevents PD-L1 degradation, further enhancing PD-L1 protein expression on the cells surface. In T cells, NFAT activity is restrained by CDK4/6-mediated phosphorylation. Following CDK4/6 inhibition, hypophosphorylated NFAT translocates to the nucleus and upregulates expression of effector genes. Compared to other lymphocyte populations, T regulatory cells appear particularly susceptible to the anti-proliferative effects of CDK4/6 inhibition.

One of the most well-defined mechanisms by which CDK4/6i augments anti-tumor immunity is through enhancing the immunogenicity of tumor cells. Most notably, CDK4/6i increases expression of antigen presenting genes (H2d1, H2k1, B2m, Erap1, Tap1, Tap2) and surface expression of MHC I and MHC II in several mouse and human breast and colon carcinoma pre-clinical models (108, 109, 113) (**Figure 3**). Likewise, elevated *CCND1* expression (encoding the CDK4/6 binding partner, Cyclin D1) is associated with lower expression of MHC I genes in breast cancer patients (108, 114). Less is known about the effects of CDK4/6 inhibition on melanoma cells, however, an increase in MHC I in the mouse melanoma cell line B16-OVA in response to CDK4/6 inhibition has been reported, which led to enhanced T cell recognition *in vitro* (108), In breast cancer preclinical models and patients, induction of tumor-intrinsic interferon signaling is observed in response to CDK4/6i, which may enhance tumor immunogenicity by promoting increased secretion of T cell chemoattractants and expression of costimulatory genes (108) (**Figure 3**). This increased interferon signaling is reportedly due to suppression of DNA methyltransferase, DNMT1, (an E2F target gene), which reduces methylation of endogenous retroviral genes, thereby promoting their expression and inducing viral mimicry (108) (**Figure 3**).

Interestingly, tumor immunogenicity in response to CDK4/6i is not attributable to the induction of senescence. Cellular senescence is a state of irreversible cell cycle arrest that initiates a senescent-associated secretory phenotype (SASP), typified by the secretion of pro-inflammatory cytokines that recruit immune cells (109, 115, 116). While CDK4/6i increases B-galactosidase staining (indicative of senescence), no other SASP genes are induced, suggesting that senescence does not contribute to CDK4/6i-induced tumor immunogenicity (108, 109). In addition to their immune-potentiating effects, inhibitors of CDK4/6 can also dampen immunity *via* increased expression of PD-L1 on tumor cells (109, 110, 112, 113). PD-L1 fluctuates throughout the cell cycle and is lowest during late G1-S when CDK4/6 is most active (110). Inhibition of CDK4/6, or likewise knocking out Cyclin D1 or overexpressing p16^INK4A^, leads to increased PD-L1 expression in numerous cell lines and preclinical tumor models, including B16 melanoma (109, 110, 112, 113).

#### T Cell-Intrinsic Effects of CDK4/6 Inhibition

While the majority of studies examining the immunomodulatory activity of CDK4/6 inhibition focused on tumor-intrinsic effects or effects on the tumor microenvironment more broadly, a seminal article in 2018 reported on the direct impact of CDK4/6 inhibition on T cells (111). In this study, Deng and colleagues hit CDK4/6 inhibitors in a small molecule screen aimed at identifying enhancers of T cell activation, using IL-2 production as a readout, and subsequently identified NFAT as a novel phosphorylation substrate of CDK6 in T cells (111). Upon CDK4/6 inhibition, phosphorylation of NFAT is prevented, leading to its de-repression and subsequent translocation to the nucleus where it promotes the transcription of effector response genes (111) (**Figure 3**). Indeed, increased NFAT signaling occurs in tumors following CDK4/6 inhibition (109), and an increase in IFNγ production by T cells in response to CDK4/6 inhibition has also been reported (108, 111). In addition to enhancing T cell activation, NFAT transcription factors can also promote T cell exhaustion. Interestingly however, exhaustion markers appear to be decreased in T cells following CDK4/6 inhibition, though this has not been fully explored (108, 111). In fact, the direct effects of CDK4/6 inhibition on T cell phenotype and function is entirely unknown. Given these cells are the primary effectors of anti-tumor immunity and immunotherapies, this is an area of considerable interest.

In addition to the regulation of NFAT, CDK4/6 may also play a role in T cell proliferation, however the mechanisms are not well defined. In human T cells, CDK4/6i has modest effects on proliferation and no impact on viability *in vitro* (109). Within the tumor microenvironment, the effects of CDK4/6i on the T cell infiltrate appear to vary depending on the tumor model and treatment schedule used. In MMTV-Erbb2 breast, MC38 colon, and B16-F10 melanoma tumor models, CDK4/6 inhibition led to a decrease in total numbers of CD3+ tumor infiltrating T cells, encompassing CD8^+^ T cells and Granzyme B^+^ and IFNγ^+^ cells (110). In contrast, in the CT26 colon carcinoma model, CD3^+^ T cell numbers were relatively unchanged following CDK4/6 inhibition (109). While some studies have demonstrated an increase in the frequency of CD3^+^ T cells in the immune compartment of tumors (108, 109), it is unclear whether this is a true increase in the number of T cells, or simply a consequence of other immune populations shifting. Overall, it appears that CDK4/6 inhibition leads to less or equal absolute numbers of CD3^+^ T cells in the tumor microenvironment (109, 110), suggesting a possible anti-proliferative effect of CDK4/6i on these cells. Interestingly, in a syngeneic breast cancer model, CDK4/6 inhibition led to a significant reduction in the frequency and absolute number of tumor-infiltrating CD4^+^CD25^+^ Tregs, while other T cell subsets were unaffected (108), suggesting that Tregs are the most susceptible T cell subset to the anti-proliferative effects of CDK4/6i. This reduction in Tregs was attributed to cytostasis, as no changes were observed in apoptosis or the production of Tregs in the thymus (108).

Given the scarcity of studies that have investigated the direct effects of CDK4/6 inhibition on T cells, the role of CDK4 and CDK6 in these cells can be gleaned from studies that have utilized transgenic mice deficient for these kinases. Indeed, T cells from CDK6-null mice demonstrate a significant delay in proliferation and RB phosphorylation following mitogenic stimulation, indicating an important role for CDK6 in T cell proliferation following activation (117). Early studies also hinted at a role for CDK4 in T cell proliferation, as low levels of CDK4 expression correlated with reduced T cell proliferation (118). However, thymocytes from CDK4^-/-^ bone marrow chimeric mice show no defects in proliferation or cytokine production (119), though interestingly, CDK4^-/-^ mice had underdeveloped thymuses and significantly increased numbers of CD8/CD4 negative thymocytes (119). A decrease in thymic mass and an increase in CD4^+^CD8^+^ thymocytes has also be observed in response to CDK4/6 inhibition (108), suggesting CDK4 may play a role in T cell development and maturation.

## Targeted Therapy And Immune Checkpoint Blockade Combinations

The prospect of combining targeted therapies with ICB, either concurrently or consecutively, for the treatment of melanoma is attractive for two main reasons. Firstly, the clinical response profiles of these therapies are distinct, in that targeted therapies provide short-term benefits for a majority of patients, while ICB provides long-term benefits for a minority of patients. Assuming an additive effect, this combination strategy therefore has the potential to both increase the number of patients that respond clinically and extend the therapeutic benefit for some. Secondly, given the profound impact targeted therapies have on anti-tumor immunity, these drugs may synergize with ICB by creating a tumor microenvironment that is more conducive to supporting a clinically beneficial long-term response to immunotherapy. Pre-clinical studies and clinical trials examining MAPK/ERK- or CDK4/6-targeted therapy in combination with ICB will be discussed in the following sections.

### Combining MAPK/ERK-Targeted Therapy With Immune Checkpoint Blockade

A number of pre-clinical studies have shown promising results from combining BRAF or BRAF and MEK inhibition with ICB for the treatment of melanoma. In mouse models of BRAF^V600^ melanoma, blockade of either PD-1 or PD-L1 synergized with BRAF inhibitor monotherapy (93) or dual inhibition of BRAF and MEK (106, 120, 121). This effect was dependent on CD8^+^ T cells (121), and attributed to increased numbers and function of tumor infiltrating lymphocytes seen in response to the combination (93). Additionally, resistance to BRAF inhibitors is associated with increased expression of PD-L1 by tumor cells, further suggesting PD-1 blockade would synergize with BRAF inhibition (122). Surprisingly, preclinical studies examining the efficacy of MAPK/ERK inhibition with CTLA-4 blockade in melanoma are lacking. Interestingly however, CTLA-4 blockade synergizes with BRAF inhibition in BRAF-wildtype colon carcinoma and fibrosarcoma mouse models (123); an effect attributed to the expansion of antigen specific T cells resulting from BRAFi-induced paradoxical activation of MAPK/ERK (123). Additionally, continual treatment of BRAFi- or BRAFi/MEKi-resistant tumors with BRAFi renders tumor cells more susceptible to killing by cytotoxic lymphocytes through transiently enhancing uptake of the T cell cytotoxic molecule, granzyme B (124). Notably, these studies suggest that the combined benefits of BRAF inhibition with T cell-directed immunotherapies may extend beyond BRAF-mutant or BRAFi-sensitive cancers.

Retrospective clinical data can provide insight into the potential efficacy of combining targeted therapy and ICB. Indeed, retrospective analysis from two clinical studies found that patients who progressed on BRAFi and MEKi, and subsequently went on to receive anti-PD-1 or anti-CTLA-4 therapy, had poor overall response rates (125, 126), possibly due to the loss of TILs associated with resistance to MAPK/ERK inhibition (93, 94). However, there are case reports where anti-CTLA-4 therapy has shown efficacy after BRAF inhibition (93, 127). Whether prior BRAF inhibition induced sensitivity to anti-CTLA-4 therapy, or whether these tumors were inherently sensitive to begin with was unclear.

Despite the conflicting data from these retrospective cases, the success of this combination in pre-clinical studies led to the initiation of several clinical trials designed to prospectively assess the efficacy of combined MAPK/ERK targeted therapy and ICB (reviewed in 128–130). Early phase safety trials of MAPK/ERK inhibitors co-administered with anti-CTLA-4 therapy demonstrated significant toxicities (131–133). Interestingly, toxicity was associated with the addition of trametinib (MEKi), and appeared worse for vemurafenib (BRAFi) than dabrafenib (BRAFi) (133), highlighting the need to consider specific drug-related adverse events in the design of combination therapies. BRAF/MEK-targeted therapies in combination with anti-PD-1 or anti-PD-L1 therapies have shown better overall toxicity profiles than that of anti-CTLA-4 therapy, however trial results have been variable. A randomized phase III trial (IMspire170) examining the benefit of adding a MEK inhibitor to PD-L1 blockade showed disappointing results, with the combination of cobimetinib (MEKi) and atezolizumab (anti-PD-L1) failing to increase PFS compared to pembrolizumab (anti-PD-1) alone (134). Trials examining the triplet combination of dual BRAF/MEK-targeted therapy and PD-(L)1 blockade have been more encouraging. Of note, in the phase II trial, KEYNOTE-022, dual dabrafenib/trametinib plus pembrolizumab showed a numerical increase in PFS compared to dabrafenib/trametinib plus placebo (16 vs.10.3 months); however, this did not reach statistical significance (135, 136). More recently, two seminal phase III trials of triplet BRAF/MEK/PD-(L)1 therapy (COMBI-I and TRILOGY IMspire150) yielded conflicting results (137–139). The primary outcome measured in both trials was investigator-assessed PFS comparing dual BRAFi/MEKi/PD-(L)1 versus BRAFi/MEKi/placebo, and while IMspire150 successfully met its primary endpoint, COMBI-I failed to do so. Similar to the KEYNOTE-022 trial, results from COMBI-I were nonetheless encouraging, demonstrating a trend in increased PFS with the addition of the PD-1 inhibitor, spartalizumab, to dabrafenib/trametinib (16.2 vs 12 months) (139), but falling short of statistical significance. In contrast, IMspire150 reported a statistical benefit with the addition of the PD-L1 inhibitor, atezolizumab, to dual vemurafenib/cobimetinib (15.1 vs. 10.6 months) (137). The details and results of these trials are summarized in **Table 1**.

The overall design of the COMBI-I and IMspire150 trials were similar, drawing into question the conflicting results of the primary outcome from these two trials. A key difference was the use of a run-in cycle of BRAFi/MEKi prior to the addition of ICB in the IMspire150 trial, compared to upfront administration of the triplet synchronously in the COMBI-I trial (137, 139). Given the profound immunomodulatory effects of BRAFi/MEKi described above, this run-in may alter tumor susceptibility to subsequent ICB dosing. Also of note, the PD-1 inhibitor, sparatlizumab, used in COMBI-I, is a newly developed monoclonal antibody. While it is unlikely this inhibitor is less efficacious than other PD-(L)1 inhibitors, it has not yet proven efficacious in clinical trials, and thus its utility in combination therapies should be interpreted with caution. An additional consideration in these trials is the baseline characteristics of patients undergoing therapy. For example, factors such as elevated lactate dehydrogenase and greater number of disease sites at baseline predicts worse overall prognosis for patients treated with targeted therapy (140). Patients with poorer prognosis in this regard may demonstrate superior benefit from the addition of anti-PD-(L)1 therapy, compared to those with baseline characteristics associated with better responses to dual BRAF/MEK-targeted therapy. Notably, patients in the dual BRAFi/MEKi arm of COMBI-I did better than those in the dual arm of IMspire150 (12 vs 10.6 months), potentially due to a lower proportion of patients in the COMBI-I trial with more than three disease sites at baseline (45% in COMBI-I vs. 56% in IMspire150; (**Table 1**) (137, 139). Indeed, a higher PFS in the dual BRAFi/MEKi arm of COMBI-I compared to IMspire150 may have been a determining factor differentiating the statistical outcomes of these trials.

**Table 1:** Design, results and key features of major clinical trials evaluating BRAF/MEK/PD-(L)1 triple combination therapy.

	KEYNOTE-022				COMBI-I				IMspire150			
**Trial ID**	NCT02130466				NCT02967692				NCT02908672			
**Reference**	(136)				(139)				(137)			
**Trial type**	Double blind, randomized, placebo-controlled, Phase I/II				Double blind, randomized, placebo-controlled, Phase III				Double blind, randomized, placebo-controlled, Phase III			
**Therapeutic**	Pembrolizumab (Keytruda^®^)	Dabrafenib (TAFINLAR^®^)		Trametinib (Mekinist^®^)	Spartalizumab	Dabrafenib (TAFINLAR^®^)		Trametinib (Mekinist^®^)	Atezolizumab (TECENTRIQ^®^)	Vemurafenib (Zelbolraf^®^)		Cobimetinib (COTELLIC^®^)
**Target**	PD-1	BRAF		MEK	PD-1	BRAF		MEK	PD-L1	BRAF		MEK
**Schedule**	Triplet used up front				Triplet used up front				One cycle run-in with vemurafenib + cobimetinib only, followed by triplet in subsequent cycles			
**Cohort**	Patients with previously untreated BRAFV600 melanoma (unresectable, locally advanced or metastatic)				Patients with previously untreated BRAFV600 melanoma (unresectable, locally advanced or metastatic)				Patients with previously untreated BRAFV600 melanoma (unresectable, locally advanced or metastatic)			
**Primary outcome assessed**	Investigator-assessed PFS				Investigator-assessed PFS				Investigator-assessed PFS			

**Trial arms**	Pembrolizumab + dabrafenib + trametinib		Placebo + dabrafenib + trametinib		Spartalizumab + dabrafenib + trametinib		Placebo + dabrafenib + trametinib		Atezolizumab + vemurafenib + cobimetinib		Placebo + vemurafenib + cobimetinib	
**Number of patients**	60		60		267		265		256		258	
**Median follow up (months)**	9.6				27.2				18.9			
**PFS (months)**	**16**		**10.3**		**16.2**		**12**		**15.1**		**10.6**	
	HR, 0.66 [95% CI,0.40-10.7]; p= 0.043 **Not significant**				HR, 0.82 [95% CI, 0.655-1.027]; p=0.042 **Not significant**				HR, 0.78 [95% CI, 0.63-0.97]; p=0·025 **Significant**			
**ORR (%)**	63.3		71.7		68.5		64.2		66.3		65	
**CRR (%)**	18.3		13.3		20		18		15.7		17.1	
**Tx-related adverse events requiring discontinuation (%)**	25		15		12		8		13		16	
**Baseline characteristics (% of patients)**												
**Elevated LDH**	45		43.3		~40				33		33	
**Metastasis stage M1c**	81.7		63.3		~60				57		63	
**Disease at >2-3 sites**	63.3 (>2)		53.3 (>2)		~45 (>3)				56 (>3)		56 (>3)	
**Previously treated for CNS metastases**	1.7		1.7						2		3	

While the primary outcome measured in IMspire150 and COMBI-I was investigator-assessed PFS, IMspire150 also included assessment of PFS by an independent review committee. Notably, in contrast to the investigator assessment, independent review determined there was no statistically significant increase in PFS upon the addition of atezolizumab to vemurafenib/cobimetinib (16.1 vs 12.3 months) (137). Interestingly, the IMspire150 results from the independent assessment align with those from the COMBI-I trial. As such, the conflicting results in these trials may be due more to statistical design and analysis of the trial, rather than differences in the clinical outcome of the combinations. Encouragingly, toxicities were manageable in both trials and the consistent numerical increase in PFS is promising. Adjustments in dose and schedule, or alternative targeted therapy/ICB combinations, may therefore have potential to improve outcomes, and many further clinical trials are ongoing in this space (NCT02224781, NCT04511013, NCT03554083, NCT04310397, NCT02910700). Further, the capacity for BRAF/MEK-targeted therapies to enhance immunotherapies other than ICB, such as high-dose IL-2, oncolytic viral therapies and adoptive cell therapy, including CAR-T cell therapy, requires further exploration in the clinic. Such approaches have shown promise in preclinical and small-scale clinical studies, and may present additional avenues for the utility of BRAF/MEK inhibitors as immunotherapy adjuvants (106, 124, 141, 142).

### Combining CDK4/6 Inhibition With Immune Checkpoint Blockade

The recently discovered immunomodulatory activity of CDK4/6i have made it an attractive potential adjuvant for T cell directed immunotherapies. Indeed, CDK4/6 inhibitors have demonstrated synergy with blockade of the PD-1/PD-L1 axis in a number of preclinical models (108–111), prompting evaluation of this combination therapy in clinical trials. Pre-clinically, this combination is particularly efficacious against CT26 colon carcinoma cells (108–110), which interestingly harbor a *Cdkn2a* deletion and expresses functional RB (143, 144), likely rendering it highly sensitive to the tumor-intrinsic immunomodulatory effects of CDK4/6 inhibition. In this model, the most efficacious schedule was treatment with a CDK4/6 inhibitor daily from days 6-34, with anti-PD-L1 antibody administration on days 13, 20 & 27 (109). The resulting anti-tumor efficacy of this combination schedule was attributed to CDK4/6 inhibition promoting and maintaining a T cell inflamed microenvironment (109). Efficacy of this combination has been demonstrated in a number of other pre-clinical models, including MC38 colon carcinoma (109, 110), MMTV-Her2 breast cancer (108), EMT6 (109), as well as AT3-OVA breast cancer with the addition of a PI3K inhibitor (113). Interestingly, CDK4/6 inhibition promotes PD-L1 surface expression on tumor cells through both RB-dependent and RB-independent mechanisms (110, 112). This suggests that CDK4/6i-induced effects on tumor PD-L1 expression, and subsequent synergy with anti-PD1/PD-L1 therapy, may also be relevant for RB-deficient tumor types.

As CDK4/6 inhibitors and PD-1/PD-L1 checkpoint inhibitors are already developed for clinical use, the combination of these two therapies has been able to rapidly enter clinical trials. In light of encouraging pre-clinical data, several clinical trials are now underway to examine the safety and efficacy of this combination. These trials are being conducted predominantly in ER^+^ breast cancer, as well as other advanced solid tumors (NCT04075604, NCT02778685, NCT04118036, NCT03147287, NCT03294694, NCT02791334). As this combination is new to the clinic, examining the toxicity profile will be a crucial first step and results from these trials are eagerly anticipated. In the interim, the immunomodulatory effects of CDK4/6 inhibitors are still being elucidated and additional pre-clinical studies are required to better understand how to most effectively use CDK4/6 inhibitors to boost anti-tumor immunity.

## Conclusion

The past decade has seen a rapid rise in the introduction of modern therapies that have transformed treatment outcomes for patients with melanoma. Dominating this landscape has been the development of small-molecule targeted therapies and ICB for the treatment of unresectable or metastatic melanoma. Individually, both targeted therapies and ICB continue to produce remarkable clinical responses in melanoma patients, but the challenge is to overcome the caveats that arise from acquired resistance and poor response rates to these therapies. As a result, numerous clinical trials are underway to explore new targeted therapy combinations, such as BRAF, MEK and CDK4/6 inhibition, as well as combination targeted therapy and ICB approaches, designed with the aim of capitalizing on the distinct mechanistic modes of action of these therapies. As these latter combinations enter the clinic, it is imperative that we continue to investigate the complex and time-dependent immunomodulatory effects of targeted therapies and develop a thorough understanding of how these agents impact on the tumor immune microenvironment and anti-tumor immune responses. It is clear targeted therapies have dynamic multi-faceted effects on anti-tumor immunity, both indirectly *via* interruption of tumor intrinsic pathways that modulate immune responses, and directly through modulating these signaling pathways within immune cells themselves. Our appreciation of the complexities of the immune composition of tumors is growing rapidly in an era of advanced single cell and high-throughput multi-omic technology, providing us with new understanding of the immune subsets required for optimal responses to ICB. The goal now is to incorporate this evolving knowledge to gain a deeper understanding of how targeted therapies influence these immune cell subsets, and identify how this can be exploited to augment anti-tumor immunity and responses to ICB. Such knowledge is essential to facilitate the rational design of combinations and scheduling regimes of targeted and immune-based therapies that maximize positive outcomes for patients.

## Author Contributions

EL conceptualized and wrote the manuscript and created the figures. GM, JO, and KS provided feedback and reviewed the manuscript. All authors contributed to the article and approved the submitted version.

## Funding

This research was supported by the Peter MacCallum Cancer Foundation and grants from National Health and Medical Research Council (NHMRC) to GM & KS (1100189, 1175894) and JO (1139626); National Breast Cancer Foundation grant to JO (IIRS-18-151); Peter Mac Postgraduate Scholarship, Melbourne University Research Scholarship (58616) and Cancer Therapeutics CRC PhD Top Up Scholarship to EL.

## Conflict of Interest

The authors declare that the research was conducted in the absence of any commercial or financial relationships that could be construed as a potential conflict of interest.

## References

[1] Gandini S Sera F Cattaruzza MS Pasquini P Abeni D Boyle P. Meta-Analysis of Risk Factors for Cutaneous Melanoma: I. Common and Atypical Naevi. Eur J Cancer (2005) 41:28–44. 10.1016/j.ejca.2004.10.015 15617989

[2] Markovic SN Erickson LA Rao RD Weenig RH Pockaj BA Bardia A. Malignant Melanoma in the 21st Century, Part 1: Epidemiology, Risk Factors, Screening, Prevention, and Diagnosis. Mayo Clin Proc (2007) 82:364–80. 10.4065/82.3.364 17352373

[3] Mackie RM Hauschild A Eggermont AM. Epidemiology of Invasive Cutaneous Melanoma. Ann Oncol (2009) 20:vi1 7. 10.1093/annonc/mdp252 PMC2712590 19617292

[4] De Vries E Houterman S Janssen-Heijnen ML Nijsten T Van De Schans SA Eggermont AM. Up-to-Date Survival Estimates and Historical Trends of Cutaneous Malignant Melanoma in the South-East of the Netherlands. Ann Oncol (2007) 18:1110–6. 10.1093/annonc/mdm087 17434898

[5] American Cancer Society. Survival Rates for Melanoma Skin Cancer (2020) (Accessed June 2020).

[6] Song X Zhao Z Barber B Farr AM Ivanov B Novich M. Overall Survival in Patients With Metastatic Melanoma. Curr Med Res Opin (2015) 31:987–91. 10.1185/03007995.2015.1021904 25708472

[7] Robert C Grob JJ Stroyakovskiy D Karaszewska B Hauschild A Levchenko E. Five-Year Outcomes With Dabrafenib Plus Trametinib in Metastatic Melanoma. N Engl J Med (2019) 381:626–36. 10.1056/NEJMoa1904059 31166680

[8] Ascierto PA Dummer R Gogas HJ Flaherty KT Arance A Mandala M. Update on Tolerability and Overall Survival in COLUMBUS: Landmark Analysis of a Randomised Phase 3 Trial of Encorafenib Plus Binimetinib Vs Vemurafenib or Encorafenib in Patients With BRAF V600-Mutant Melanoma. Eur J Cancer (2020) 126:33–44. 10.1016/j.ejca.2019.11.016 31901705

[9] Ribas A Daud A Pavlick AC Gonzalez R Lewis KD Hamid O. Extended 5-Year Follow-Up Results of a Phase Ib Study (BRIM7) of Vemurafenib and Cobimetinib in BRAF-Mutant Melanoma. Clin Cancer Res (2020) 26:46–53. 10.1158/1078-0432.CCR-18-4180 31732523 PMC6942621

[10] Ribas A Hamid O Daud A Hodi FS Wolchok JD Kefford R. Association of Pembrolizumab With Tumor Response and Survival Among Patients With Advanced Melanoma. JAMA (2016) 315:1600–9. 10.1001/jama.2016.4059 27092830

[11] Larkin J Chiarion-Sileni V Gonzalez R Grob JJ Rutkowski P Lao CD. Five-Year Survival With Combined Nivolumab and Ipilimumab in Advanced Melanoma. N Engl J Med (2019) 381:1535–46. 10.1056/NEJMoa1910836 31562797

[12] Robert C Ribas A Schachter J Arance A Grob JJ Mortier L. Pembrolizumab Versus Ipilimumab in Advanced Melanoma (KEYNOTE-006): Post-Hoc 5-Year Results From an Open-Label, Multicentre, Randomised, Controlled, Phase 3 Study. Lancet Oncol (2019) 20:1239–51. 10.1016/S1470-2045(19)30388-2 31345627

[13] Hodi FS O’day SJ Mcdermott DF Weber RW Sosman JA Haanen JB. Improved Survival With Ipilimumab in Patients With Metastatic Melanoma. N Engl J Med (2010) 363:711–23. 10.1056/NEJMoa1003466 PMC3549297 20525992

[14] Robert C Thomas L Bondarenko I O’day S Weber J Garbe C. Ipilimumab Plus Dacarbazine for Previously Untreated Metastatic Melanoma. N Engl J Med (2011) 364:2517–26. 10.1056/NEJMoa1104621 21639810

[15] Robert C Long GV Brady B Dutriaux C Maio M Mortier L. Nivolumab in Previously Untreated Melanoma Without BRAF Mutation. N Engl J Med (2015) 372:320–30. 10.1056/NEJMoa1412082 25399552

[16] Wang DY Salem JE Cohen JV Chandra S Menzer C Ye F. Fatal Toxic Effects Associated With Immune Checkpoint Inhibitors: A Systematic Review and Meta-Analysis. JAMA Oncol (2018) 4:1721–8. 10.1001/jamaoncol.2018.3923 PMC6440712 30242316

[17] Chai QQ Du JY Zhu J Wu B. The Differences in the Safety and Tolerability of Immune Checkpoint Inhibitors as Treatment for Non-Small Cell Lung Cancer and Melanoma: Network Meta-Analysis and Systematic Review. Front Pharmacol (2019) 10:1260. 10.3389/fphar.2019.01260 31708783 PMC6821878

[18] Verma V Sprave T Haque W Simone CB,2 Chang JY Welsh JW. A Systematic Review of the Cost and Cost-Effectiveness Studies of Immune Checkpoint Inhibitors. J Immunother Cancer (2018) 6:128. 10.1186/s40425-018-0442-7 30470252 PMC6251215

[19] Wang HT Choi B Tang MS. Melanocytes are Deficient in Repair of Oxidative DNA Damage and UV-Induced Photoproducts. Proc Natl Acad Sci USA (2010) 107:12180–5. 10.1073/pnas.1005244107 PMC2901481 20566850

[20] Chow AY. Cell Cycle Control by Oncogenes and Tumor Suppressors: Driving the Transformation of Normal Cells Into Cancerous Cells. Nat Education (2010) 3:7.

[21] Weinstein IB. Cancer. Addiction to Oncogenes–the Achilles Heal of Cancer. Science (2002) 297:63–4. 10.1126/science.1073096 12098689

[22] Davies H Bignell GR Cox C Stephens P Edkins S Clegg S. Mutations of the BRAF Gene in Human Cancer. Nature (2002) 417:949–54. 10.1038/nature00766 12068308

[23] Garnett MJ Marais R. Guilty as Charged: B-RAF is a Human Oncogene. Cancer Cell (2004) 6:313–9. 10.1016/j.ccr.2004.09.022 15488754

[24] Cantwell-Dorris ER O’leary JJ Sheils OM. BRAFV600E: Implications for Carcinogenesis and Molecular Therapy. Mol Cancer Ther (2011) 10:385–94. 10.1158/1535-7163.MCT-10-0799 21388974

[25] Colombino M Capone M Lissia A Cossu A Rubino C De Giorgi V. BRAF/NRAS Mutation Frequencies Among Primary Tumors and Metastases in Patients With Melanoma. J Clin Oncol (2012) 30:2522–9. 10.1200/JCO.2011.41.2452 22614978

[26] Wan PT Garnett MJ Roe SM Lee S Niculescu-Duvaz D Good VM. Mechanism of Activation of the RAF-ERK Signaling Pathway by Oncogenic Mutations of B-RAF. Cell (2004) 116:855–67. 10.1016/s0092-8674(04)00215-6 15035987

[27] Downward J. Targeting RAS Signalling Pathways in Cancer Therapy. Nat Rev Cancer (2003) 3:11–22. 10.1038/nrc969 12509763

[28] Cancer Genome Atlas N. Genomic Classification of Cutaneous Melanoma. Cell (2015) 161:1681–96. 10.1016/j.cell.2015.05.044 PMC4580370 26091043

[29] Sensi M Nicolini G Petti C Bersani I Lozupone F Molla A. Mutually Exclusive NRASQ61R and BRAFV600E Mutations At the Single-Cell Level in the Same Human Melanoma. Oncogene (2006) 25:3357–64. 10.1038/sj.onc.1209379 16462768

[30] Smalley I Smalley KSM. Erk Inhibition: A New Front in the War Against MAPK Pathway-Driven Cancers? Cancer Discovery (2018) 8:140–2. 10.1158/2159-8290.CD-17-1355 29431672

[31] Savoia P Fava P Casoni F Cremona O. Targeting the ERK Signaling Pathway in Melanoma. Int J Mol Sci (2019) 20. 10.3390/ijms20061483 PMC6472057 30934534

[32] Tsai J Lee JT Wang W Zhang J Cho H Mamo S. Discovery of a Selective Inhibitor of Oncogenic B-Raf Kinase With Potent Antimelanoma Activity. Proc Natl Acad Sci USA (2008) 105:3041–6. 10.1073/pnas.0711741105 PMC2268581 18287029

[33] Chapman PB Hauschild A Robert C Haanen JB Ascierto P Larkin J. Improved Survival With Vemurafenib in Melanoma With BRAF V600E Mutation. N Engl J Med (2011) 364:2507–16. 10.1056/NEJMoa1103782 PMC3549296 21639808

[34] Banzi M De Blasio S Lallas A Longo C Moscarella E Alfano R. Dabrafenib: A New Opportunity for the Treatment of BRAF V600-Positive Melanoma. Onco Targets Ther (2016) 9:2725–33. 10.2147/OTT.S75104 PMC4866744 27226731

[35] Koelblinger P Thuerigen O Dummer R. Development of Encorafenib for BRAF-Mutated Advanced Melanoma. Curr Opin Oncol (2018) 30:125–33. 10.1097/CCO.0000000000000426 PMC5815646 29356698

[36] Chapman PB. Mechanisms of Resistance to RAF Inhibition in Melanomas Harboring a BRAF Mutation. Am Soc Clin Oncol Educ Book (2013) 33:e80–82. 10.1200/EdBook_AM.2013.33.e80 23714462

[37] Luebker SA Koepsell SA. Diverse Mechanisms of BRAF Inhibitor Resistance in Melanoma Identified in Clinical and Preclinical Studies. Front Oncol (2019) 9:268. 10.3389/fonc.2019.00268 31058079 PMC6478763

[38] Flaherty KT Infante JR Daud A Gonzalez R Kefford RF Sosman J. Combined BRAF and MEK Inhibition in Melanoma With BRAF V600 Mutations. N Engl J Med (2012) 367:1694–703. 10.1056/NEJMoa1210093 PMC3549295 23020132

[39] Flaherty KT Robert C Hersey P Nathan P Garbe C Milhem M. Improved Survival With MEK Inhibition in BRAF-Mutated Melanoma. N Engl J Med (2012) 367:107–14. 10.1056/NEJMoa1203421 22663011

[40] Matsushime H Ewen ME Strom DK Kato JY Hanks SK Roussel MF. Identification and Properties of an Atypical Catalytic Subunit (P34psk-J3/Cdk4) for Mammalian D Type G1 Cyclins. Cell (1992) 71:323–34. 10.1016/0092-8674(92)90360-O 1423597

[41] Giacinti C Giordano A. RB and Cell Cycle Progression. Oncogene (2006) 25:5220–7. 10.1038/sj.onc.1209615 16936740

[42] Burkhart DL Sage J. Cellular Mechanisms of Tumour Suppression by the Retinoblastoma Gene. Nat Rev Cancer (2008) 8:671–82. 10.1038/nrc2399 PMC6996492 18650841

[43] Sherr CJ. Cancer Cell Cycles. Science. (1996) 274:1672–7. 10.1126/science.274.5293.1672 8939849

[44] Dyson N. The Regulation of E2F by Prb-Family Proteins. Genes Dev (1998) 12:2245–62. 10.1101/gad.12.15.2245 9694791

[45] Ewen ME Sluss HK Sherr CJ Matsushime H Kato J Livingston DM. Functional Interactions of the Retinoblastoma Protein With Mammalian D-Type Cyclins. Cell (1993) 73:487–97. 10.1016/0092-8674(93)90136-E 8343202

[46] Kato J Matsushime H Hiebert SW Ewen ME Sherr CJ. Direct Binding of Cyclin D to the Retinoblastoma Gene Product (Prb) and Prb Phosphorylation by the Cyclin D-Dependent Kinase CDK4. Genes Dev (1993) 7:331–42. 10.1101/gad.7.3.331 8449399

[47] Hanahan D Weinberg RA. Hallmarks of Cancer: The Next Generation. Cell (2011) 144:646–74. 10.1016/j.cell.2011.02.013 21376230

[48] Serrano M Hannon GJ Beach D. A New Regulatory Motif in Cell-Cycle Control Causing Specific Inhibition of Cyclin D/CDK4. Nature (1993) 366:704–7. 10.1038/366704a0 8259215

[49] Walker GJ Flores JF Glendening JM Lin AH Markl ID Fountain JW. Virtually 100% of Melanoma Cell Lines Harbor Alterations At the DNA Level Within CDKN2A, CDKN2B, or One of Their Downstream Targets. Genes Chromosomes Cancer (1998) 22:157–63. 10.1002/(sici)1098-2264(199806)22:2<157::aid-gcc11>3.0.co;2-n 9598804

[50] Curtin JA Fridlyand J Kageshita T Patel HN Busam KJ Kutzner H. Distinct Sets of Genetic Alterations in Melanoma. N Engl J Med (2005) 353:2135–47. 10.1056/NEJMoa050092 16291983

[51] Sheppard KE Mcarthur GA. The Cell-Cycle Regulator CDK4: An Emerging Therapeutic Target in Melanoma. Clin Cancer Res (2013) 19:5320–8. 10.1158/1078-0432.CCR-13-0259 24089445

[52] Hall M Peters G. Genetic Alterations of Cyclins, Cyclin-Dependent Kinases, and Cdk Inhibitors in Human Cancer. Adv Cancer Res (1996) 68:67–108. 10.1016/S0065-230X(08)60352-8 8712071

[53] Knudsen ES Knudsen KE. Tailoring to RB: Tumour Suppressor Status and Therapeutic Response. Nat Rev Cancer (2008) 8:714–24. 10.1038/nrc2401 PMC2914856 19143056

[54] Schwartz GK Lorusso PM Dickson MA Randolph SS Shaik MN Wilner KD. Phase I Study of PD 0332991, a Cyclin-Dependent Kinase Inhibitor, Administered in 3-Week Cycles (Schedule 2/1). Br J Cancer (2011) 104:1862–8. 10.1038/bjc.2011.177 PMC3111206 21610706

[55] Turner NC Huang Bartlett C Cristofanilli M. Palbociclib in Hormone-Receptor-Positive Advanced Breast Cancer. N Engl J Med (2015) 373:1672–3. 10.1056/NEJMc1510345 26488700

[56] Sherr CJ Beach D Shapiro GI. Targeting CDK4 and CDK6: From Discovery to Therapy. Cancer Discovery (2016) 6:353–67. 10.1158/2159-8290.CD-15-0894 PMC4821753 26658964

[57] Mebratu Y Tesfaigzi Y. How ERK1/2 Activation Controls Cell Proliferation and Cell Death: is Subcellular Localization the Answer? Cell Cycle (2009) 8:1168–75. 10.4161/cc.8.8.8147 PMC2728430 19282669

[58] Lavoie JN L’allemain G Brunet A Muller R Pouyssegur J. Cyclin D1 Expression is Regulated Positively by the P42/P44mapk and Negatively by the P38/HOGMAPK Pathway. J Biol Chem (1996) 271:20608–16. 10.1074/jbc.271.34.20608 8702807

[59] Terada Y Nakashima O Inoshita S Kuwahara M Sasaki S Marumo F. Mitogen-Activated Protein Kinase Cascade and Transcription Factors: The Opposite Role of MKK3/6-P38k and MKK1-MAPK. Nephrol Dial Transplant (1999) 14:45–7. 10.1093/ndt/14.suppl_1.45 10048449

[60] Cheng M Sexl V Sherr CJ Roussel MF. Assembly of Cyclin D-Dependent Kinase and Titration of p27Kip1 Regulated by Mitogen-Activated Protein Kinase Kinase (MEK1). Proc Natl Acad Sci USA (1998) 95:1091–6. 10.1073/pnas.95.3.1091 PMC18683 9448290

[61] Smalley KS Lioni M Dalla Palma M Xiao M Desai B Egyhazi S. Increased Cyclin D1 Expression Can Mediate BRAF Inhibitor Resistance in BRAF V600E-Mutated Melanomas. Mol Cancer Ther (2008) 7:2876–83. 10.1158/1535-7163.MCT-08-0431 PMC2651569 18790768

[62] Flaherty KD Davies MA Grob JJ Long GV Nathan PD Ribas A. Genomic Analysis and 3-Y Efficacy and Safety Update of COMBI-D: A Phase 3 Study of Dabrafenib (D) + Trametinib (T) Vs D Monotherapy in Patients (Pts) With Unresectable or Metastatic BRAF V600E/K-Mutant Cutaneous Melanoma. J Clin Oncol (2016) 34:Abstr 9502. 10.1200/JCO.2016.34.15_suppl.9502

[63] Yadav V Burke TF Huber L Van Horn RD Zhang Y Buchanan SG. The CDK4/6 Inhibitor LY2835219 Overcomes Vemurafenib Resistance Resulting From MAPK Reactivation and Cyclin D1 Upregulation. Mol Cancer Ther (2014) 13:2253–63. 10.1158/1535-7163.MCT-14-0257 25122067

[64] Yoshida A Lee EK Diehl JA. Induction of Therapeutic Senescence in Vemurafenib-Resistant Melanoma by Extended Inhibition of CDK4/6. Cancer Res (2016) 76:2990–3002. 10.1158/0008-5472.CAN-15-2931 26988987 PMC4873417

[65] Martin CA Cullinane C Kirby L Abuhammad S Lelliott EJ Waldeck K. Palbociclib Synergizes With BRAF and MEK Inhibitors in Treatment Naive Melanoma But Not After the Development of BRAF Inhibitor Resistance. Int J Cancer (2018) 142:2139–52. 10.1002/ijc.31220 29243224

[66] Lelliott EJ Mangiola S Ramsbottom KM Zethoven M Lim L Lau PKH. Combined BRAF, MEK, and CDK4/6 Inhibition Depletes Intratumoral Immune-Potentiating Myeloid Populations in Melanoma. Cancer Immunol Res (2021) 9:136–46. 10.1158/2326-6066.CIR-20-0401 33303574

[67] Lee MS Helms TL Feng N Gay J Chang QE Tian F. Efficacy of the Combination of MEK and CDK4/6 Inhibitors in Vitro and in Vivo in KRAS Mutant Colorectal Cancer Models. Oncotarget (2016) 7:39595–608. 10.18632/oncotarget.9153 PMC5129956 27167191

[68] Kwong LN Costello JC Liu H Jiang S Helms TL Langsdorf AE. Oncogenic NRAS Signaling Differentially Regulates Survival and Proliferation in Melanoma. Nat Med (2012) 18:1503–10. 10.1038/nm.2941 PMC3777533 22983396

[69] Posch C Sanlorenzo M Ma J Kim ST Zekhtser M Ortiz-Urda S. MEK/CDK4,6 Co-Targeting is Effective in a Subset of NRAS, BRAF and ‘Wild Type’ Melanomas. Oncotarget (2018) 9:34990–5. 10.18632/oncotarget.26204 PMC6201855 30405888

[70] Teh JLF Cheng PF Purwin TJ Nikbakht N Patel P Chervoneva I. In Vivo E2f Reporting Reveals Efficacious Schedules of MEK1/2-CDK4/6 Targeting and Mtor-S6 Resistance Mechanisms. Cancer Discovery (2018) 8:568–81. 10.1158/2159-8290.CD-17-0699 PMC6858088 29496664

[71] Ascierto PA Bechter O Wolter P Lebbe C Elez E Miller WH. A Phase Ib/II Dose-Escalation Study Evaluating Triple Combination Therapy With a BRAF (Encorafenib), MEK (Binimetinib), and CDK 4/6 (Ribociclib) Inhibitor in Patients (Pts) With BRAF V600-Mutant Solid Tumors and Melanoma. J Clin Oncol (2017) 35. 10.1200/JCO.2017.35.15_suppl.9518

[72] Raedler LA. Opdivo (Nivolumab): Second Pd-1 Inhibitor Receives FDA Approval for Unresectable or Metastatic Melanoma. Am Health Drug Benefits (2015) 8:180–3. PMC4665056 26629287

[73] Yu JH Hodge JP Olivia C Neftelinov ST Hubbard-Lucey VM Tang J. Trends in Clinical Development for PD-1/PD-L1 Inhibitors. Nat Rev Drug Discovery (2020) 19:163–4. 10.1038/d41573-019-00182-w 32127660

[74] Wei SC Duffy CR Allison JP. Fundamental Mechanisms of Immune Checkpoint Blockade Therapy. Cancer Discovery (2018) 8:1069–86. 10.1158/2159-8290.CD-18-0367 30115704

[75] Homet Moreno B Parisi G Robert L Ribas A. Anti-PD-1 Therapy in Melanoma. Semin Oncol (2015) 42:466–73. 10.1053/j.seminoncol.2015.02.008 25965365

[76] Lafleur MW Muroyama Y Drake CG Sharpe AH. Inhibitors of the PD-1 Pathway in Tumor Therapy. J Immunol (2018) 200:375–83. 10.4049/jimmunol.1701044 PMC5924692 29311378

[77] Schachter J Ribas A Long GV Arance A Grob JJ Mortier L. Pembrolizumab Versus Ipilimumab for Advanced Melanoma: Final Overall Survival Results of a Multicentre, Randomised, Open-Label Phase 3 Study (KEYNOTE-006). Lancet (2017) 390:1853–62. 10.1016/S0140-6736(17)31601-X 28822576

[78] Larkin J Chiarion-Sileni V Gonzalez R Grob JJ Cowey CL Lao CD. Combined Nivolumab and Ipilimumab or Monotherapy in Untreated Melanoma. N Engl J Med (2015) 373:23–34. 10.1056/NEJMoa1504030 26027431 PMC5698905

[79] Postow MA Chesney J Pavlick AC Robert C Grossmann K Mcdermott D. Nivolumab and Ipilimumab Versus Ipilimumab in Untreated Melanoma. N Engl J Med (2015) 372:2006–17. 10.1056/NEJMoa1414428 PMC5744258 25891304

[80] Rowshanravan B Halliday N Sansom DM. Ctla-4: A Moving Target in Immunotherapy. Blood (2018) 131:58–67. 10.1182/blood-2017-06-741033 29118008 PMC6317697

[81] Tivol EA Boyd SD Mckeon S Borriello F Nickerson P Strom TB. CTLA4Ig Prevents Lymphoproliferation and Fatal Multiorgan Tissue Destruction in CTLA-4-Deficient Mice. J Immunol (1997) 158:5091–4. 9164923

[82] Wing K Onishi Y Prieto-Martin P Yamaguchi T Miyara M Fehervari Z. Ctla-4 Control Over Foxp3+ Regulatory T Cell Function. Science (2008) 322:271–5. 10.1126/science.1160062 18845758

[83] Tai X Van Laethem F Pobezinsky L Guinter T Sharrow SO Adams A. Basis of CTLA-4 Function in Regulatory and Conventional CD4(+) T Cells. Blood (2012) 119:5155–63. 10.1182/blood-2011-11-388918 PMC3369608 22403258

[84] Ha D Tanaka A Kibayashi T Tanemura A Sugiyama D Wing JB. Differential Control of Human Treg and Effector T Cells in Tumor Immunity by Fc-Engineered Anti-CTLA-4 Antibody. Proc Natl Acad Sci USA (2019) 116:609–18. 10.1073/pnas.1812186116 PMC6329979 30587582

[85] Yang YF Zou JP Mu J Wijesuriya R Ono S Walunas T. Enhanced Induction of Antitumor T-Cell Responses by Cytotoxic T Lymphocyte-Associated Molecule-4 Blockade: The Effect is Manifested Only At the Restricted Tumor-Bearing Stages. Cancer Res (1997) 57:4036–41. 9307290

[86] Van Elsas A Hurwitz AA Allison JP. Combination Immunotherapy of B16 Melanoma Using Anti-Cytotoxic T Lymphocyte-Associated Antigen 4 (CTLA-4) and Granulocyte/Macrophage Colony-Stimulating Factor (GM-CSF)-Producing Vaccines Induces Rejection of Subcutaneous and Metastatic Tumors Accompanied by Autoimmune Depigmentation. J Exp Med (1999) 190:355–66. 10.1084/jem.190.3.355 PMC2195583 10430624

[87] Yost KE Satpathy AT Wells DK Qi Y Wang C Kageyama R. Clonal Replacement of Tumor-Specific T Cells Following PD-1 Blockade. Nat Med (2019) 25:1251–9. 10.1038/s41591-019-0522-3 PMC6689255 31359002

[88] Chow MT Ozga AJ Servis RL Frederick DT Lo JA Fisher DE. Intratumoral Activity of the CXCR3 Chemokine System is Required for the Efficacy of Anti-PD-1 Therapy. Immunity (2019) 50:1498–512.e5. 10.1016/j.immuni.2019.04.010 31097342 PMC6527362

[89] House IG Savas P Lai J Chen AXY Oliver AJ Teo ZL. Macrophage-Derived CXCL9 and CXCL10 are Required for Antitumor Immune Responses Following Immune Checkpoint Blockade. Clin Cancer Res (2020) 26:487–504. 10.1158/1078-0432.CCR-19-1868 31636098

[90] Sumimoto H Imabayashi F Iwata T Kawakami Y. The BRAF-MAPK Signaling Pathway is Essential for Cancer-Immune Evasion in Human Melanoma Cells. J Exp Med (2006) 203:1651–6. 10.1084/jem.20051848 PMC2118331 16801397

[91] Sapkota B Hill CE Pollack BP. Vemurafenib Enhances MHC Induction in BRAFV600E Homozygous Melanoma Cells. Oncoimmunology (2013) 2:e22890. 10.4161/onci.22890 23483066 PMC3583938

[92] Ilieva KM Correa I Josephs DH Karagiannis P Egbuniwe IU Cafferkey MJ. Effects of BRAF Mutations and BRAF Inhibition on Immune Responses to Melanoma. Mol Cancer Ther (2014) 13:2769–83. 10.1158/1535-7163.MCT-14-0290 PMC4258403 25385327

[93] Wilmott JS Long GV Howle JR Haydu LE Sharma RN Thompson JF. Selective BRAF Inhibitors Induce Marked T-Cell Infiltration Into Human Metastatic Melanoma. Clin Cancer Res (2012) 18:1386–94. 10.1158/1078-0432.CCR-11-2479 22156613

[94] Frederick DT Piris A Cogdill AP Cooper ZA Lezcano C Ferrone CR. BRAF Inhibition is Associated With Enhanced Melanoma Antigen Expression and a More Favorable Tumor Microenvironment in Patients With Metastatic Melanoma. Clin Cancer Res (2013) 19:1225–31. 10.1158/1078-0432.CCR-12-1630 PMC3752683 23307859

[95] Cooper ZA Juneja VR Sage PT Frederick DT Piris A Mitra D. Response to BRAF Inhibition in Melanoma is Enhanced When Combined With Immune Checkpoint Blockade. Cancer Immunol Res (2014) 2:643–54. 10.1158/2326-6066.CIR-13-0215 PMC4097121 24903021

[96] Boni A Cogdill AP Dang P Udayakumar D Njauw CN Sloss CM. Selective BRAFV600E Inhibition Enhances T-Cell Recognition of Melanoma Without Affecting Lymphocyte Function. Cancer Res (2010) 70:5213–9. 10.1158/0008-5472.CAN-10-0118 20551059

[97] Heidorn SJ Milagre C Whittaker S Nourry A Niculescu-Duvas I Dhomen N. Kinase-Dead BRAF and Oncogenic RAS Cooperate to Drive Tumor Progression Through CRAF. Cell (2010) 140:209–21. 10.1016/j.cell.2009.12.040 PMC2872605 20141835

[98] Poulikakos PI Zhang C Bollag G Shokat KM Rosen N. RAF Inhibitors Transactivate RAF Dimers and ERK Signalling in Cells With Wild-Type BRAF. Nature (2010) 464:427–30. 10.1038/nature08902 PMC3178447 20179705

[99] Tse A Verkhivker GM. Exploring Molecular Mechanisms of Paradoxical Activation in the BRAF Kinase Dimers: Atomistic Simulations of Conformational Dynamics and Modeling of Allosteric Communication Networks and Signaling Pathways. PloS One (2016) 11:e0166583. 10.1371/journal.pone.0166583 27861609 PMC5115767

[100] Koya RC Mok S Otte N Blacketor KJ Comin-Anduix B Tumeh PC. BRAF Inhibitor Vemurafenib Improves the Antitumor Activity of Adoptive Cell Immunotherapy. Cancer Res (2012) 72:3928–37. 10.1158/0008-5472.CAN-11-2837 PMC3422880 22693252

[101] Ferrari De Andrade L Ngiow SF Stannard K Rusakiewicz S Kalimutho M Khanna KK. Natural Killer Cells are Essential for the Ability of BRAF Inhibitors to Control BRAFV600E-Mutant Metastatic Melanoma. Cancer Res (2014) 74:7298–308. 10.1158/0008-5472.CAN-14-1339 25351955

[102] Wang T Xiao M Ge Y Krepler C Belser E Lopez-Coral A. Braf Inhibition Stimulates Melanoma-Associated Macrophages to Drive Tumor Growth. Clin Cancer Res (2015) 21:1652–64. 10.1158/1078-0432.CCR-14-1554 PMC4383683 25617424

[103] Chapman PB Solit DB Rosen N. Combination of RAF and MEK Inhibition for the Treatment of BRAF-Mutated Melanoma: Feedback is Not Encouraged. Cancer Cell (2014) 26:603–4. 10.1016/j.ccell.2014.10.017 25517746

[104] Vella LJ Pasam A Dimopoulos N Andrews M Knights A Puaux AL. MEK Inhibition, Alone or in Combination With BRAF Inhibition, Affects Multiple Functions of Isolated Normal Human Lymphocytes and Dendritic Cells. Cancer Immunol Res (2014) 2:351–60. 10.1158/2326-6066.CIR-13-0181 24764582

[105] Ebert PJ Cheung J Yang Y Mcnamara E Hong R Moskalenko M. Map Kinase Inhibition Promotes T Cell and Anti-Tumor Activity in Combination With PD-L1 Checkpoint Blockade. Immunity (2016) 44:609–21. 10.1016/j.immuni.2016.01.024 26944201

[106] Hu-Lieskovan S Mok S Homet Moreno B Tsoi J Robert L Goedert L. Improved Antitumor Activity of Immunotherapy With BRAF and MEK Inhibitors in BRAF(V600E) Melanoma. Sci Transl Med (2015) 7:279ra41. 10.1126/scitranslmed.aaa4691 PMC4765379 25787767

[107] Erkes DA Cai W Sanchez IM Purwin TJ Rogers C Field CO. Mutant BRAF and MEK Inhibitors Regulate the Tumor Immune Microenvironment Via Pyroptosis. Cancer Discovery (2020) 10:254–69. 10.1158/2159-8290.CD-19-0672 PMC7007378 31796433

[108] Goel S Decristo MJ Watt AC Brinjones H Sceneay J Li BB. Cdk4/6 Inhibition Triggers Anti-Tumour Immunity. Nature (2017) 548:471–5. 10.1038/nature23465 PMC5570667 28813415

[109] Deng J Wang ES Jenkins RW Li S Dries R Yates K. Cdk4/6 Inhibition Augments Antitumor Immunity by Enhancing T-Cell Activation. Cancer Discovery (2018) 8:216–33. 10.1158/2159-8290.CD-17-0915 PMC5809273 29101163

[110] Schaer DA Beckmann RP Dempsey JA Huber L Forest A Amaladas N. The CDK4/6 Inhibitor Abemaciclib Induces a T Cell Inflamed Tumor Microenvironment and Enhances the Efficacy of PD-L1 Checkpoint Blockade. Cell Rep (2018) 22:2978–94. 10.1016/j.celrep.2018.02.053 29539425

[111] Zhang J Bu X Wang H Zhu Y Geng Y Nihira NT. Cyclin D-CDK4 Kinase Destabilizes PD-L1 Via Cullin 3-SPOP to Control Cancer Immune Surveillance. Nature (2018) 553:91–5. 10.1038/nature25015 PMC5754234 29160310

[112] Jin X Ding D Yan Y Li H Wang B Ma L. Phosphorylated RB Promotes Cancer Immunity by Inhibiting NF-Kappab Activation and PD-L1 Expression. Mol Cell (2019) 73:22–35.e6. 10.1016/j.molcel.2018.10.034 30527665 PMC8968458

[113] Teo ZL Versaci S Dushyanthen S Caramia F Savas P Mintoff CP. Combined CDK4/6 and PI3Kalpha Inhibition is Synergistic and Immunogenic in Triple-Negative Breast Cancer. Cancer Res (2017) 77:6340–52. 10.1158/0008-5472.CAN-17-2210 28947417

[114] Cerami E Gao J Dogrusoz U Gross BE Sumer SO Aksoy BA. The Cbio Cancer Genomics Portal: An Open Platform for Exploring Multidimensional Cancer Genomics Data. Cancer Discovery (2012) 2:401–4. 10.1158/2159-8290.CD-12-0095 PMC3956037 22588877

[115] Coppe JP Desprez PY Krtolica A Campisi J. The Senescence-Associated Secretory Phenotype: The Dark Side of Tumor Suppression. Annu Rev Pathol (2010) 5:99–118. 10.1146/annurev-pathol-121808-102144 20078217 PMC4166495

[116] Munoz-Espin D Serrano M. Cellular Senescence: From Physiology to Pathology. Nat Rev Mol Cell Biol (2014) 15:482–96. 10.1038/nrm3823 24954210

[117] Malumbres M Sotillo R Santamaria D Galan J Cerezo A Ortega S. Mammalian Cells Cycle Without the D-Type Cyclin-Dependent Kinases Cdk4 and Cdk6. Cell (2004) 118:493–504. 10.1016/j.cell.2004.08.002 15315761

[118] Brunner MC Chambers CA Chan FK Hanke J Winoto A Allison JP. Ctla-4-Mediated Inhibition of Early Events of T Cell Proliferation. J Immunol (1999) 162:5813–20. 10229815

[119] Chow YH Zhu XD Liu L Schwartz BR Huang XZ Harlan JM. Role of Cdk4 in Lymphocyte Function and Allergen Response. Cell Cycle (2010) 9:4922–30. 10.4161/cc.9.24.14209 PMC3047815 21150327

[120] Deken MA Gadiot J Jordanova ES Lacroix R Van Gool M Kroon P. Targeting the MAPK and PI3K Pathways in Combination With PD1 Blockade in Melanoma. Oncoimmunology (2016) 5:e1238557. 10.1080/2162402X.2016.1238557 28123875 PMC5215252

[121] Homet Moreno B Mok S Comin-Anduix B Hu-Lieskovan S Ribas A. Combined Treatment With Dabrafenib and Trametinib With Immune-Stimulating Antibodies for BRAF Mutant Melanoma. Oncoimmunology (2016) 5:e1052212. 10.1080/2162402X.2015.1052212 27622011 PMC5006894

[122] Jiang X Zhou J Giobbie-Hurder A Wargo J Hodi FS. The Activation of MAPK in Melanoma Cells Resistant to BRAF Inhibition Promotes PD-L1 Expression That is Reversible by MEK and PI3K Inhibition. Clin Cancer Res (2013) 19:598–609. 10.1158/1078-0432.CCR-12-2731 23095323

[123] Callahan MK Masters G Pratilas CA Ariyan C Katz J Kitano S. Paradoxical Activation of T Cells Via Augmented ERK Signaling Mediated by a RAF Inhibitor. Cancer Immunol Res (2014) 2:70–9. 10.1158/2326-6066.CIR-13-0160 PMC3883307 24416731

[124] Atay C Kwak T Lavilla-Alonso S Donthireddy L Richards A Moberg V. Braf Targeting Sensitizes Resistant Melanoma to Cytotoxic T Cells. Clin Cancer Res (2019) 25:2783–94. 10.1158/1078-0432.CCR-18-2725 PMC6497575 30765391

[125] Ackerman A Klein O Mcdermott DF Wang W Ibrahim N Lawrence DP. Outcomes of Patients With Metastatic Melanoma Treated With Immunotherapy Prior to or After BRAF Inhibitors. Cancer (2014) 120:1695–701. 10.1002/cncr.28620 24577748

[126] Simeone E Grimaldi AM Festino L Giannarelli D Vanella V Palla M. Correlation Between Previous Treatment With BRAF Inhibitors and Clinical Response to Pembrolizumab in Patients With Advanced Melanoma. Oncoimmunology (2017) 6:e1283462. 10.1080/2162402X.2017.1283462 28405510 PMC5384373

[127] Hassel JC Lee SB Meiss F Meier F Dimitrakopoulou-Strauss A Jager D. Vemurafenib and Ipilimumab: A Promising Combination? Results of a Case Series. Oncoimmunology (2016) 5:e1101207. 10.1080/2162402X.2015.1101207 27141385 PMC4839308

[128] Pelster MS Amaria RN. Combined Targeted Therapy and Immunotherapy in Melanoma: A Review of the Impact on the Tumor Microenvironment and Outcomes of Early Clinical Trials. Ther Adv Med Oncol (2019) 11:1758835919830826. 10.1177/1758835919830826 30815041 PMC6384439

[129] Yu C Liu X Yang J Zhang M Jin H Ma X. Combination of Immunotherapy With Targeted Therapy: Theory and Practice in Metastatic Melanoma. Front Immunol (2019) 10:990. 10.3389/fimmu.2019.00990 31134073 PMC6513976

[130] Dummer R Ascierto PA Nathan P Robert C Schadendorf D. Rationale for Immune Checkpoint Inhibitors Plus Targeted Therapy in Metastatic Melanoma: A Review. JAMA Oncol (2020) 6:1957–66. 10.1001/jamaoncol.2020.4401 32970096

[131] Ribas A Hodi FS Callahan M Konto C Wolchok J. Hepatotoxicity With Combination of Vemurafenib and Ipilimumab. N Engl J Med (2013) 368:1365–6. 10.1056/NEJMc1302338 23550685

[132] Minor DR Puzanov I Callahan MK Hug BA Hoos A. Severe Gastrointestinal Toxicity With Administration of Trametinib in Combination With Dabrafenib and Ipilimumab. Pigment Cell Melanoma Res (2015) 28:611–2. 10.1111/pcmr.12383 PMC4744965 25996827

[133] Amin A Lawson DH Salama AK Koon HB Guthrie T,Jr. Thomas SS. Phase II Study of Vemurafenib Followed by Ipilimumab in Patients With Previously Untreated BRAF-Mutated Metastatic Melanoma. J Immunother Cancer (2016) 4:44. 10.1186/s40425-016-0148-7 27532019 PMC4986368

[134] Gogas H Dreno B Larkin J Demidov L Stroyakovskiy D Eroglu Z. Cobimetinib Plus Atezolizumab in BRAF(V600) Wild-Type Melanoma: Primary Results From the Randomized Phase III Imspire170 Study. Ann Oncol (2021) 32:384–94. 10.1016/j.annonc.2020.12.004 33309774

[135] Ribas AH Hodi FS Lawrence D Atkinson V Agarwal S Carlino MS. Keynote-022 Update: Phase 1 Study of First-Line Pembrolizumab (Pembro) Plus Dabrafenib (D) and Trametinib (T) for BRAF-Mutant Advanced Melanoma. Ann Oncol (2017) 28:v428–48 abstr 2503. 10.1093/annonc/mdx377.003

[136] Ascierto PA Ferrucci PF Fisher R Del Vecchio M Atkinson V Schmidt H. Dabrafenib, Trametinib and Pembrolizumab or Placebo in BRAF-Mutant Melanoma. Nat Med (2019) 25:941–6. 10.1038/s41591-019-0448-9 31171878

[137] Dummer RF Fernández AM Hansson J Larkin JMG Long GV Gasal E. Preliminary Findings From Part 1 of COMBI-I: A Phase III Study of Anti–PD-1 Antibody PDR001 Combined With Dabrafenib (D) and Trametinib (T) in Previously Untreated Patients (Pts) With Advanced BRAF V600-Mutant Melanoma. J Clin Oncol (2018) Abstr 189. 10.1200/JCO.2018.36.5_suppl.189

[138] Gutzmer R Stroyakovskiy D Gogas H Robert C Lewis K Protsenko S. Atezolizumab, Vemurafenib, and Cobimetinib as First-Line Treatment for Unresectable Advanced BRAF(V600) Mutation-Positive Melanoma (Imspire150): Primary Analysis of the Randomised, Double-Blind, Placebo-Controlled, Phase 3 Trial. Lancet (2020) 395:1835–44. 10.1016/S0140-6736(20)30934-X 32534646

[139] Nathan P Dummer R Long GV Ascierto PA Tawbi HA Robert C. Lba43 - Spartalizumab Plus Dabrafenib and Trametinib (Sparta-Dabtram) in Patients (Pts) With Previously Untreated BRAF V600–Mutant Unresectable or Metastatic Melanoma: Results From the Randomized Part 3 of the Phase III COMBI-I Trial. Ann Oncol (2020) 31. 10.1016/j.annonc.2020.08.2273

[140] Long GV Grob JJ Nathan P Ribas A Robert C Schadendorf D. Factors Predictive of Response, Disease Progression, and Overall Survival After Dabrafenib and Trametinib Combination Treatment: A Pooled Analysis of Individual Patient Data From Randomised Trials. Lancet Oncol (2016) 17:1743–54. 10.1016/S1470-2045(16)30578-2 27864013

[141] Bommareddy PK Aspromonte S Zloza A Rabkin SD Kaufman HL. MEK Inhibition Enhances Oncolytic Virus Immunotherapy Through Increased Tumor Cell Killing and T Cell Activation. Sci Transl Med (2018) 10. 10.1126/scitranslmed.aau0417 PMC7593827 30541787

[142] Mooradian MJ Reuben A Prieto PA Hazar-Rethinam M Frederick DT Nadres B. A Phase II Study of Combined Therapy With a BRAF Inhibitor (Vemurafenib) and Interleukin-2 (Aldesleukin) in Patients With Metastatic Melanoma. Oncoimmunology (2018) 7:e1423172. 10.1080/2162402X.2017.1423172 29721378 PMC5927481

[143] Finn RS Dering J Conklin D Kalous O Cohen DJ Desai AJ. PD 0332991, a Selective Cyclin D Kinase 4/6 Inhibitor, Preferentially Inhibits Proliferation of Luminal Estrogen Receptor-Positive Human Breast Cancer Cell Lines in Vitro. Breast Cancer Res (2009) 11:R77. 10.1186/bcr2419 19874578 PMC2790859

[144] Castle JC Loewer M Boegel S De Graaf J Bender C Tadmor AD. Immunomic, Genomic and Transcriptomic Characterization of CT26 Colorectal Carcinoma. BMC Genomics (2014) 15:190. 10.1186/1471-2164-15-190 24621249 PMC4007559

